# Lagrangian Particle Dispersion Models in the Grey Zone of Turbulence: Adaptations to FLEXPART-COSMO for Simulations at 1 km Grid Resolution

**DOI:** 10.1007/s10546-022-00728-3

**Published:** 2022-08-05

**Authors:** Ioannis Katharopoulos, Dominik Brunner, Lukas Emmenegger, Markus Leuenberger, Stephan Henne

**Affiliations:** 1 Laboratory for Air Pollution/Environmental Technology, Swiss Federal Laboratories for Materials Science and Technology (Empa), Dübendorf, Switzerland; 2 Institute for Atmospheric and Climate Science, ETH Zürich, Zurich, Switzerland; 3 Physics Institute, Climate and Environmental Physics, University of Bern, Bern, Switzerland; 4 Oeschger Centre for Climate Change Research, Bern, Switzerland

**Keywords:** Boundary layer, Greenhouse gas emissions, Inverse modelling, Lagrangian transport modelling, Turbulence

## Abstract

Lagrangian particle dispersion models (LPDMs) are frequently used for regional-scale inversions of greenhouse gas emissions. However, the turbulence parameterizations used in these models were developed for coarse resolution grids, hence, when moving to the kilometre-scale the validity of these descriptions should be questioned. Here, we analyze the influence of the turbulence parameterization employed in the LPDM FLEXPART-COSMO model. Comparisons of the turbulence kinetic energy between the turbulence schemes of FLEXPART-COSMO and the underlying Eulerian model COSMO suggest that the dispersion in FLEXPART-COSMO suffers from a double-counting of turbulent elements when run at a high resolution of $1 \times 1 \,\hbox {km}^2$. Such turbulent elements are represented in both COSMO, by the resolved grid-scale winds, and FLEXPART, by its stochastic parameterizations. Therefore, we developed a new parametrization for the variations of the winds and the Lagrangian time scales in FLEXPART in order to harmonize the amount of turbulence present in both models. In a case study for a power plant plume, the new scheme results in improved plume representation when compared with in situ flight observations and with a tracer transported in COSMO. Further in-depth validation of the LPDM against methane observations at a tall tower site in Switzerland shows that the model’s ability to predict the observed tracer variability and concentration at different heights above ground is considerably enhanced using the updated turbulence description. The high-resolution simulations result in a more realistic and pronounced diurnal cycle of the tracer concentration peaks and overall improved correlation with observations when compared to previously used coarser resolution simulations (at 7 km $\times$ 7 km). Our results indicate that the stochastic turbulence schemes of LPDMs, developed in the past for coarse resolution models, should be revisited to include a resolution dependency and resolve only the part of the turbulence spectrum that is a subgrid process at each different mesh size. Although our new scheme is specific to COSMO simulations at $1 \times 1 \,\hbox {km}^2$ resolution, the methodology for deriving the scheme can easily be applied to different resolutions and other regional models.

**Supplementary Information:**

The online version contains supplementary material available at 10.1007/s10546-022-00728-3.

## Introduction

Atmospheric Lagrangian particle dispersion models (LPDM) like the FLEXible PARTicle dispersion model (FLEXPART) (Stohl et al. 2005) and Met Office’s Numerical Atmospheric-dispersion Modelling Environment (NAME) (Jones et al. 2007) were first developed in the years after the nuclear accident at Chernobyl (Thomson 1987) to estimate the mesoscale and synoptic dispersion of radio-nuclei from point sources. LPDMs have evolved in the last two decades into a tool for atmospheric dispersion modelling of various atmospheric compounds ranging from long-lived greenhouse gases to aerosols. One distinguished feature of LPDMs is their straightforward applicability in both forward and backward in-time simulations (Seibert and Frank 2004; Thomson and Wilson 2012), with the backward mode allowing the computation of source receptor relationships (termed here source sensitivities) (Seibert and Frank 2004). Source sensitivities give the concentration increment at the receptor that would result from an emission source anywhere in the model domain. As such, they give the direct link between concentrations and emissions, required for inverse modelling, which is more difficult to assess in Eulerian models. Due to this ability, LPDMs have become a popular and powerful tool for inverse emissions estimation on the regional scale. Other frequent applications of LPDMs include, but are not limited to, atmospheric dispersion events such as nuclear accidents, volcanic eruptions, chemical accidents, smoke from wildfires, airborne animal diseases, and air quality forecasts (Thomson and Wilson 2012; Pisso et al. 2019). ‘Concentration’ is used interchangeably throughout the text with mixing ratio and mole fraction.

Inverse modelling is a well-known top-down emission estimation method, which utilizes numerical models to inversely estimate emissions by linking observed and simulated concentrations of atmospheric compounds. Inverse emission modelling has a recognized potential for a more general usage in real-world validation of emission inventories (bottom-up) (Nisbet and Weiss 2010; Weiss and Prinn 2011; Leip et al. 2018), which, although based on internationally recognized formalism, leave room for semi-objective choices of emission factors. It is widely applied by the scientific community for different greenhouse gases from the global to facility scale (Bousquet et al. 2006; Stohl et al. 2009, 2010; Brunner et al. 2012; Thomson and Wilson 2012; Pétron et al. 2014; Bergamaschi et al. 2015; Fang and Michalak 2015; Henne et al. 2016; Rust et al. 2022).

Inverse modelling of emissions is based on three main components: atmospheric concentration observations, source sensitivities (derived from atmospheric transport models), and an optimization algorithm (inversion framework). The accuracy of the tracer transport simulation in atmospheric models plays a crucial role, since biases in the transport may directly bias emission estimates (e.g., Karion et al. 2019). In this study, we focus on the regional-scale LPDM FLEXPART-COSMO model (Henne et al. 2016; Pisso et al. 2019) and suggest modifications to its turbulence scheme to apply it at kilometre-scale resolution. FLEXPART-COSMO is a specific version of FLEXPART that was adapted to the input from the numerical weather prediction (NWP) model COSMO (Baldauf et al. 2011).

FLEXPART-COSMO is driven by the meteorological output fields from the regional-scale model COSMO, which is operated by several national weather services at resolutions down to approximately $1 \times 1 \,\hbox {km}^2$. When the term ‘resolution’ appears in the text, it always refers to the horizontal grid resolution of the driving NWP model (COSMO). At this grid spacing ($1 \times 1 \,\hbox {km}^2$) , the resolution of the NWP model is comparable to the length scales of energy-containing turbulence elements and to the height of convective atmospheric boundary layers (ABL) (order of 100–1000  m, Wyngaard 2004). As a consequence, part of the turbulent eddies is resolved explicitly, and is thus not part of a subgrid process anymore. This region has been called the grey zone of turbulence (Beare 2014; Honnert 2016; Honnert et al. 2011, 2020) as it challenges the applicability of the employed Reynolds-averaged Navier–Stokes (RANS) approach. LPDMs operating in the grey zone should acknowledge that part of the turbulence is already resolved by the driving model. This may require modifications of their current turbulence schemes, since these were often built to parameterize the three-dimensional variations of the winds at coarse resolution. Otherwise, part of the turbulence spectrum may be duplicated in the LPDM, once as part of the mean wind fields obtained from the NWP and again as part of the LPDM’s turbulence scheme (Cornwell et al. 2021).

Duplication of turbulence will lead to increased dispersion in the LPDM. Since FLEXPART is driven by the meteorological fields of COSMO, increased dispersion in the model could be either from a misrepresentation of turbulence by FLEXPART’s turbulence scheme, or by the resolved wind fields of the Eulerian model (COSMO). Few studies have explicitly investigated diffusion in COSMO. Goger et al. (2018) suggests that there is an underestimation of turbulence kinetic energy (TKE) in Alpine valleys, due to neglecting three-dimensional effects in the TKE representation in COSMO. Westerhuis et al. (2020) suggests that COSMO’s advection scheme exhibits numerical diffusion, which induces spurious vertical mixing, and that is the reason behind the rapid dissipation of fog in COSMO when compared to observations. Here, we focus on the changes needed in the turbulence schemes of FLEXPART in order to be utilized for simulations with high-resolution meteorological fields from NWP models. We are well aware though that reliable simulations with LPDMs rely on accurate representation of turbulence and grid-resolved winds in the Eulerian models driving them.

In previous studies, FLEXPART-COSMO was successfully operated at $7 \times 7 \,\hbox {km}^2$ spatial resolution, for example to inversely estimate Swiss methane ($\mathrm {CH_4}$) emissions (Henne et al. 2016), or to simulate carbon dioxide ($\mathrm {CO_2}$) concentrations (Oney et al. 2017) and $\mathrm {CH_4}$ and nitrous oxide ($\mathrm {N_{2}O}$) isotopic composition in the Netherlands and Switzerland (Röckmann et al. 2016; Harris et al. 2017; Menoud et al. 2020). All these FLEXPART-COSMO simulations were based on operational COSMO analysis fields generated by MeteoSwiss. Since 2016, MeteoSwiss has provided COSMO analysis fields for the Alpine area at a spatial resolution of approximately $7 \times 7 \,\hbox {km}^2$ (Schmidli et al. 2018; Klasa et al. 2018; Leuenberger et al. 2020). FLEXPART-COSMO simulations and, consequently, inverse modelling studies should benefit from the increased resolution. However, when comparing FLEXPART-COSMO simulations of regional $\mathrm {CH_4}$ enhancements at different spatial resolution to observations at the Swiss tall tower site Beromünster (Berhanu et al. 2015), it became apparent that the high resolution, $1 \times 1 \,\hbox {km}^2$, simulations considerably underestimated tracer peak concentrations and variability (3-hourly averages), whereas simulations at $7 \times 7 \,\hbox {km}^2$ agreed better. Although a certain degree of underestimation of peak concentrations by any atmospheric model can be expected, due to the stochastic treatment of unresolved motion, we would expect that these discrepancies get smaller at finer model resolution.

In an attempt to remedy this behaviour, the present study analyzes the TKE present in both COSMO and FLEXPART-COSMO and suggests a reformulation of FLEXPART’s turbulence description to bring it into closer agreement with COSMO. The proposed method of the turbulence scheme derivation is not limited to our FLEXPART-COSMO setup, and can be applied to any driving NWP model. The manuscript is organized as follows: Sect. 2 describes the COSMO model and the turbulence settings used by Meteoswiss, the LPDM FLEXPART-COSMO and its default turbulence scheme, the observations, and the site used to validate the LPDM’s results. Furthermore, the development of a new (scaled) turbulence scheme is detailed. In Sect. 3 we present the improved turbulence scheme of FLEXPART-COSMO at high resolution, and discuss comparisons between simulations at different resolution and with different turbulence parameterized. Finally, conclusions are provided in Sect. 4.

## Methods

### COSMO Model

COSMO is a non-hydrostatic limited-area atmospheric model. It was first designed for operational NWP by Deutscher Wetterdienst (DWD) and it is still used and developed by several national weather services including MeteoSwiss (Baldauf et al. 2011). MeteoSwiss has been operating COSMO at three different spatial resolutions: COSMO-7 with a grid spacing of 6.6 km (until October 2020), COSMO-2 with a grid spacing of 2.2 km, and COSMO-1 with a grid spacing of 1.1 km (Schmidli et al. 2018; Klasa et al. 2018; Leuenberger et al. 2020). The model’s outer domain uses initial and boundary conditions from the European Centre for Medium-range Weather Forecasts (ECMWF) Integrated Forecast System (IFS). The available meteorological fields provided by Meteoswiss have a 1-h temporal resolution at all spatial resolutions.

COSMO utilizes RANS equations for the representation of turbulent diffusion and, as operated by MeteoSwiss, employs a one-and-a-half order (level 2.5) turbulence closure with a prognostic equation for TKE, (*e*) (Mellor and Yamada 1982). TKE is used to estimate the turbulent diffusion coefficients of the Reynolds stress tensor. Local equilibrium is assumed for all velocity moments except for TKE, where advection and turbulent transport is retained. Furthermore, the boundary-layer hypothesis of horizontal homogeneity is used, hence, only vertical turbulent fluxes are taken into account in the TKE budget, resulting in negligence of all horizontal turbulent fluxes. Although the assumption of horizontally homogeneous turbulence is usually valid for flat terrain, Goger et al. (2018) show that it may under-predict turbulence intensity in complex Alpine terrain.

### FLEXPART-COSMO

The FLEXPART-COSMO LPDM is a version of FLEXPART developed by Empa and MeteoSwiss (Henne et al. 2016). In FLEXPART-COSMO, meteorological variables are directly used on the native vertical grid of the COSMO model, in contrast to the standard FLEXPART version, which interpolates from the hybrid-sigma coordinate system of ECMWF-IFS onto a terrain-following height-based coordinate system with constant height shifts up to the model top. The meteorological fields used for the simulations of FLEXPART-COSMO are fields of instantaneous model variables (winds, temperature, pressure, etc.) and accumulated fluxes (precipitation, surface heat, momentum, moisture).

In FLEXPART, transport of infinitesimally small air parcels is simulated by a simple zero acceleration scheme,

1 $$\begin{aligned} {{\textbf {X}}}(t+dt)={{\textbf {X}}}(t)+{{\textbf {v}}}({{\textbf {X}}},t)\mathrm{{d}}t, \end{aligned}$$

where ${\mathbf {X}}$ is particle’s position, ${\mathbf {v}}$ is the sum of grid scale winds, ${\mathbf {v}}_{g}$, (in our case taken from the COSMO model), turbulent wind fluctuations, $\mathbf {v'}$, and additional mesoscale wind variations, ${\mathbf {v}}_m$:

2 $$\begin{aligned} {\mathbf {v}}({\mathbf {X}},t)={\mathbf {v}}_{g}+\mathbf {v'}+\mathbf {v_m}. \end{aligned}$$

Turbulent wind fluctuations, $\mathbf {v'}$, are a stochastic process in FLEXPART, and they are derived by a Langevin equation, Eq. 5. In addition to unresolved turbulent fluctuations, $\mathbf {v'}$, larger mesoscale (but not subgrid scale) motions, which are commonly not resolved by NWP models, can be represented in LPDMs by solving an independent Langevin equation (Webster et al. 2018). In FLEXPART, these mesoscale turbulent motions are applied as an additional three-dimensional wind component, $\mathbf {v_m}$. The required Lagrangian time scale, $\tau _{m}$, is fixed as half the timestep between input fields ($\tau _{m}$ = 1800 s in our case). The mesoscale wind variances, $\sigma _{m,i}^{2}$, are calculated from the variance of the grid-resolved wind field around (space and time) the particles’ position and an additional scaling factor $\sigma _{m,i}$ = 0.16 (Stohl et al. 2009). Tests at both 1.1 km and 6.6 km COSMO resolution showed that only little dispersion is added by this mesoscale turbulence term. Because of these small differences, and since the derivation of FLEXPART’s mesoscale turbulence is not well documented, we decided to switch it off, $\mathbf {v_m}=0$, for all our FLEXPART-COSMO simulations at both 1.1 km and 6.6 km resolution.

FLEXPART-COSMO simulations can be implemented both in a forward and in a backward mode. One major application of FLEXPART-COSMO in backward mode is inverse modelling. For this purpose, particles are released from specific observational sites and traced backward in time to obtain source-receptor relationships (Seibert and Frank 2004). These represent source sensitivities, $m_{ijk}$, in units of s $\hbox {kg}^{-1}$ mol $\hbox {mol}^{-1}$ with *i*, *j* referring to horizontal grid indices and *k* to different receptors. They can be convoluted with spatial distributions of emissions $\hbox {E}_{i,j}$ (s $\hbox {kg}^{-1}$) yielding the concentration, $\chi$ (mol $\hbox {mol}^{-1}$), of a tracer at the receptor,

3 $$\begin{aligned} \chi _{k}=\sum _{i,j}m_{i,j,k}E_{i,j}+\chi _{B,k}, \end{aligned}$$

where $\chi _B$ represents a baseline (or background) concentration, which can either be taken from observations (e.g., at nearby baseline sites) or from a larger scale Eulerian model as the average concentration at the final location of the backward integrated model particles.

**Fig. 1:**
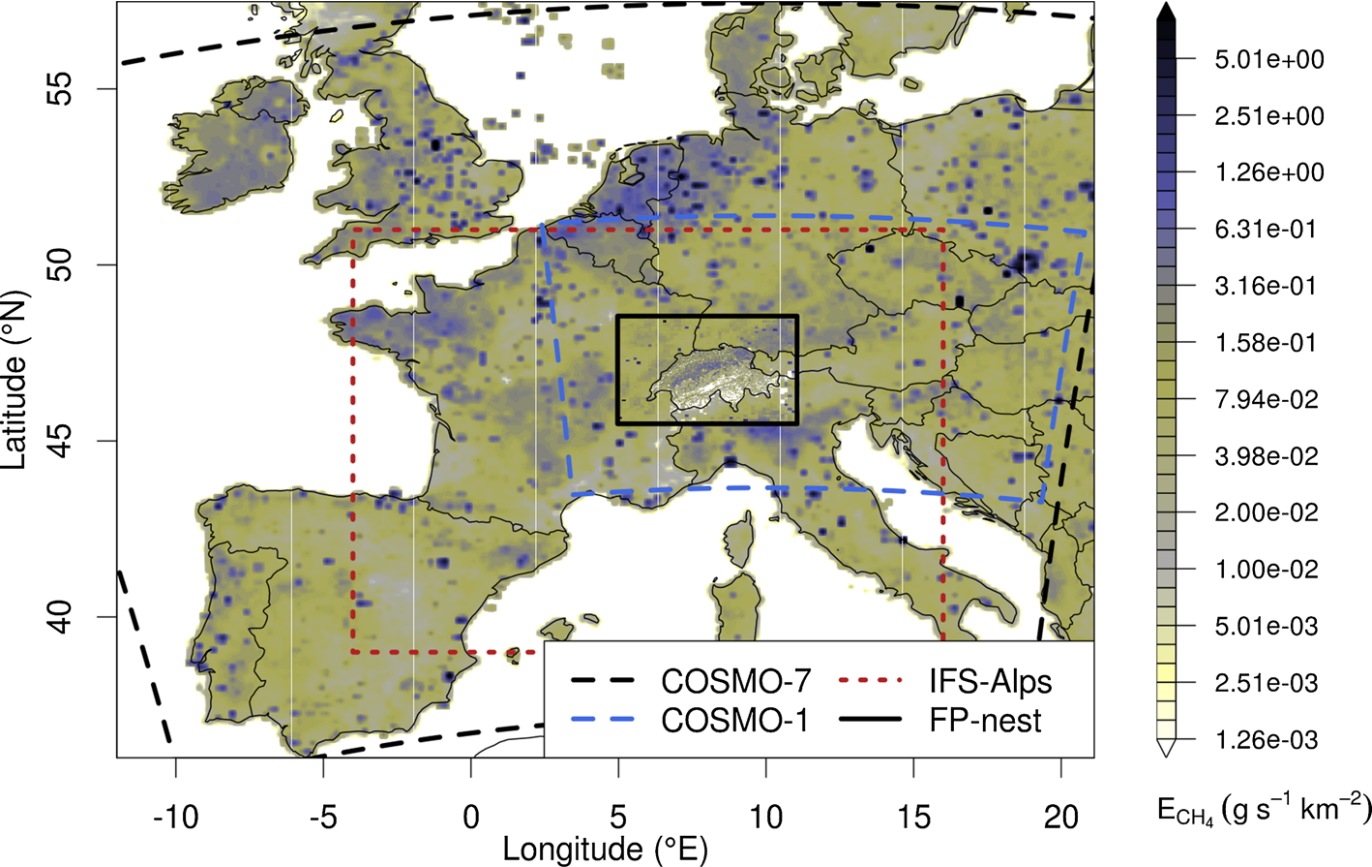
$\mathrm {CH_4}$ emissions as taken from TNO/MACC-2 and Swiss MAIOLICA inventory, merged and aggregated for the two different output grids defined for FLEPXPART-COSMO runs (main: whole figure area, nest: FP-nest). Superimposed are the two COSMO domains used as input to FLEXPART-COSMO (7 km, and 1 km) as well as the higher resolution IFS sub-domain used with FLEXPART-IFS

FLEXPART-COSMO was previously utilized at Empa employing the 7 km analysis fields provided by MeteoSwiss (COSMO-7). The COSMO-7 model domain covers part of central and western Europe ($-10^\circ$ to 20$^\circ$ E and 38$^\circ$ to 55$^\circ$ N; Fig. 1). The higher resolution operational domains of MeteoSwiss focus on the Alpine area and are considerably smaller.

If model particles were only integrated within the small high-resolution domain, some of the observed enhancements above baseline contributed by emissions outside this domain would be missed. To account for this limitation, simulations using COSMO-1 input can be nested into a larger scale domain. This can either be the COSMO-7 domain (feature readily available in FLEXPART-COSMO) or input from another NWP. Since MeteoSwiss stopped the operational production of analysis fields for the COSMO-7 domain in 2020, we decided to follow a more general approach. We offline-coupled FLEXPART-COSMO to FLEXPART-IFS allowing for continued integration of model particles in the global (or nested) IFS domain, once they leave the limited COSMO domain. For this purpose, we release particles within the FLEXPART-COSMO-1 domain, follow them until they leave the domain or until the end of the defined integration period, and store their final positions on disk. A subsequent FLEXPART-IFS simulation is then ‘re-started’ from the stored particle positions. The offline coupling implies that particles that re-enter the COSMO-1 domain continue integration in FLEXPART-IFS. Once both simulations are completed, the respective source sensitivities can be readily added to yield a single source sensitivity map. Here, FLEXPART-IFS was driven with 3-hourly analysis fields with a spatial resolution of 0.2$^\circ$ $\times$ 0.2$^\circ$ for a Central European domain (IFS-Alps in Fig. 1) and 1$^\circ$ $\times$ 1$^\circ$ elsewhere.

Turbulence in FLEXPART is parameterized as a stochastic Markov chain process following the Langevin equation, expressing the turbulent motion of wind component *i* as:

4 $$\begin{aligned} \mathrm{{d}}v_{t_{i}} = a_{i}({{\textbf {x}}},{{\textbf {v}}}_{t},t)\mathrm{{d}}t+b_{ij} ({{\textbf {x}}},{{\textbf {v}}}_{t},t)\mathrm{{d}}W_{j}, \end{aligned}$$

where *a* is the drift term and *b* the diffusion term. Both are functions of the turbulent velocity, position and time. The term $\mathrm{{d}}W_{j}$ is a Wiener process with Gaussian increments of zero mean and variance equal to $\mathrm{{d}}t$. Under the assumption that the unconditional probability density of the Eulerian velocity, *w*, at height, *z*, and time, *t*, follows a Gaussian distribution, and by applying the well-mixed criterion, the deterministic coefficient, *a*, and stochastic coefficient, *b*, can be calculated from the Fokker–Planck equation (Thomson 1987; Rodean 1996). The Langevin equation for the vertical component of the wind fluctuation, as implemented in FLEXPART, has the form:

5 $$\begin{aligned} \mathrm{{d}}w= -\frac{wdt}{\tau _{L_{w}}} + \frac{\partial \sigma _{w}^{2}}{\partial z}\mathrm{{d}}t+ \frac{\sigma _{w}^{2}}{\rho }\frac{\partial \rho }{\partial z}\mathrm{{d}}t+\left( \frac{2}{\tau _{L_{w}}}\right) ^{1/2}\sigma _{w}\mathrm{{d}}W, \end{aligned}$$

where $\sigma _{w}$ and $\tau _{L_{w}}$ are the standard deviation of the turbulent vertical wind component and the Lagrangian time scale for the vertical velocity autocorrelation. The second and the third term on the right-hand side are the drift and the density correction terms, respectively, that assure compliance with the well-mixed criterion (Stohl and Thomson 1999). The form of the drift correction term in Eq. 5 was first introduced by e.g., McNider et al. (1988), and it is a simplification of the original form (Rodean 1996), which includes an additional factor, $w^{2}/\sigma _{w}^{2}$. McNider et al. (1988) explicitly state that this form of drift correction term may lead to accumulation of particles in lower turbulence regions, a behaviour that we observe in our LPDM setup (see Sect. 3.3). Variables $\sigma _{w}$ and $\tau _{L_{w}}$ are parameterized in FLEXPART as functions of altitude above ground normalized by ABL height following similarity theory. The horizontal components of the Langevin equation are given accordingly using standard deviations of the alongwind and crosswind flow ($\sigma _{u}$, $\sigma _{v}$) and the respective Lagrangian time scales. No drift nor density correction terms are applied in the horizontal (Thomson 1987). The FLEXPART parameterization applies the suggestions of (Hanna 1982) for $\sigma _{w}$ for stable and neutral situations and those of e.g., Ryall and Maryon (1998) for unstable cases. $\tau _{L_{w}}$ is taken from (Hanna 1982) in all stability regimes. We will refer to this parametrization as Hanna (1982) in the following. The required input parameters for the Hanna (1982) scheme are ABL height, *h*; Obhukov length, *L*; convective velocity scale, $w_{*}$; and friction velocity, $u_{*}$. The term *f* corresponds to the Coriolis frequency. Please note, that for the turbulent motions, the standard deviation of the horizontal wind is derived from alongwind, $\sigma _u$, and crosswind, $\sigma _v$, components and is rotated back to geographic coordinates according to the mean wind direction at the particles’ position. For the three different stability regimes (stable, unstable, and neutral) the standard deviation of the winds are as follows:

**Neutral Conditions** ($$-1< h/L < 1$$):

6 $$\begin{aligned} \sigma _{u}= & {} 2u_{*}\exp {\left( -3fz/u_{*}\right) }, \text {and} \end{aligned}$$

7 $$\begin{aligned} \sigma _{v}= & {} \sigma _{w}=1.3u_{*}\exp {\left( -2fz/u_{*}.\right) } \end{aligned}$$

**Unstable Conditions** ($$ h/L \le -1$$):

8 $$\begin{aligned} \sigma _{v}= & {} \sigma _{u} = u_{*}\left( 12+\frac{h}{|2L|}\right) ^{1/3}, \text {and} \end{aligned}$$

9 $$\begin{aligned} \sigma _{w}= & {} \left[ 1.2\left( 1-0.9\frac{z}{h}\right) \left( \frac{z}{h}\right) ^{2/3}w_{*}^{2}+\left( 1.8-1.4\frac{z}{h}\right) u_{*}^{2}\right] ^{1/2}. \end{aligned}$$

**Stable conditions** ($$ h/L \ge 1$$):

10 $$\begin{aligned} \sigma _{u}= & {} 2u_{*}\left( 1-\frac{z}{h}\right) , \text {and} \end{aligned}$$

11 $$\begin{aligned} \sigma _{v}= & {} \sigma _{w}=1.3u_{*}\left( 1-\frac{z}{h}\right) . \end{aligned}$$

### Model Validation Target

For the validation of the LPDM, atmospheric $\mathrm {CH_4}$ observations from a tall tower site located on the Swiss Plateau were used. $\mathrm {CH_4}$ was preferred over other trace gases (carbon dioxide, carbon monoxide, nitrous oxide) for model validation, since total $\mathrm {CH_4}$ emissions and their spatial distribution in Switzerland and surrounding countries are relatively well quantified by bottom-up inventories and they undergo a relatively small diurnal and annual cycle (Henne et al. 2016). Although somewhat hilly, the Swiss plateau is relatively flat compared to the Swiss mountain ranges (Alps and Jura). It is located north of the Alps, covering about one-third of the area of Switzerland, and including about two-thirds of the population of Switzerland. Most of the Swiss $\mathrm {CH_4}$ emissions originate from that area (Hiller et al. 2014). The Beromünster (BRM) site is located roughly in the middle of the Swiss Plateau and consists of a 217-m-high decommissioned radio transmission tower. Gas inlets and meteorological instrumentation are installed on the tower at five different heights above ground (12–212 m). A comprehensive description of the installation and the measurement system can be found in Berhanu et al. (2015). For this study, $\mathrm {CH_4}$ observations from all five inlet heights were utilized, offering the unique possibility to validate simulated tracer profiles in the ABL.

Receptor-oriented FLEXPART simulations were carried out for the calculation of $\mathrm {CH_4}$ concentrations at all inlet heights for the year 2016. 50,000 particles were released at each height and continuously over each 3-h period. Particles were then traced back for 8 days for FLEXPART-COSMO-7 and for 4 and 8 days for FLEXPART-COSMO-1 coupled to FLEXPART-IFS, respectively. Source sensitivities were stored on two different output domains: a larger and coarser domain (main, 0.16$^\circ$ $\times$ 0.12$^\circ$ horizontal resolution) covering a similar area as the COSMO-7 simulations and a smaller and finer (nest, 0.02$^\circ$ $\times$ 0.015$^\circ$ horizontal resolution) domain focusing on Switzerland (Fig. 1). Output domains and resolutions were the same for all FLEXPART runs, independent of the resolution of the driving meteorology.

Finally, concentrations were calculated by applying Eq. 3 to source sensitivities calculated with different versions of FLEXPART-COSMO and $\mathrm {CH_4}$ emissions taken from the TNO/MACC-2 inventory for Europe (Kuenen et al. 2014), merged with the high-resolution Swiss MAIOLICA inventory (Fig. 1). These are the same emissions as used by Henne et al. (2016) as a priori emissions. No diurnal or annual cycle was applied to the emissions. Baseline concentrations were taken from the continuous observations at the nearby high altitude station Jungfraujoch, filtered for pollution events.

### Turbulence Kinetic Energy in COSMO and FLEXPART-COSMO

**Fig. 2:**
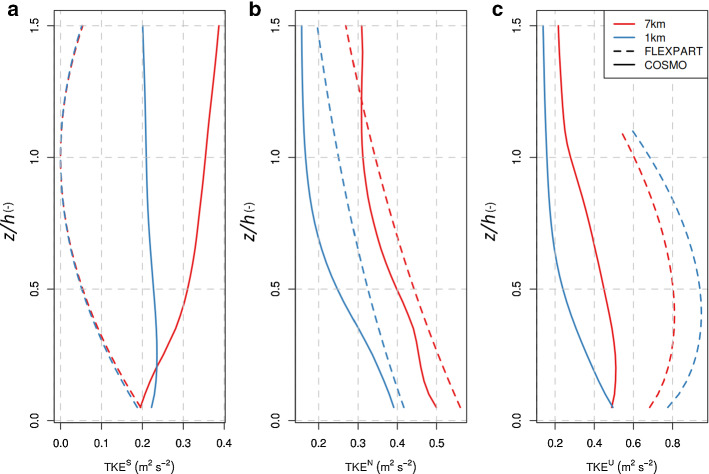
Comparisons of annual mean vertical profiles of TKE between COSMO-1 and COSMO-7 analysis fields (continuous lines correspond to COSMO’s prognostic TKE, dashed lines to diagnostic TKE from FLEXPART-COSMO): **a** stable, **b** neutral, and **c** unstable conditions ($\hbox {TKE}^S$, $\hbox {TKE}^N$, and $\hbox {TKE}^U$ respectively). The vertical axis is normalized by the ABL height. Please, note that for illustrative reasons profiles are displayed above the ABL top, although FLEXPART does not apply the turbulence scheme above the ABL top

**Fig. 3:**
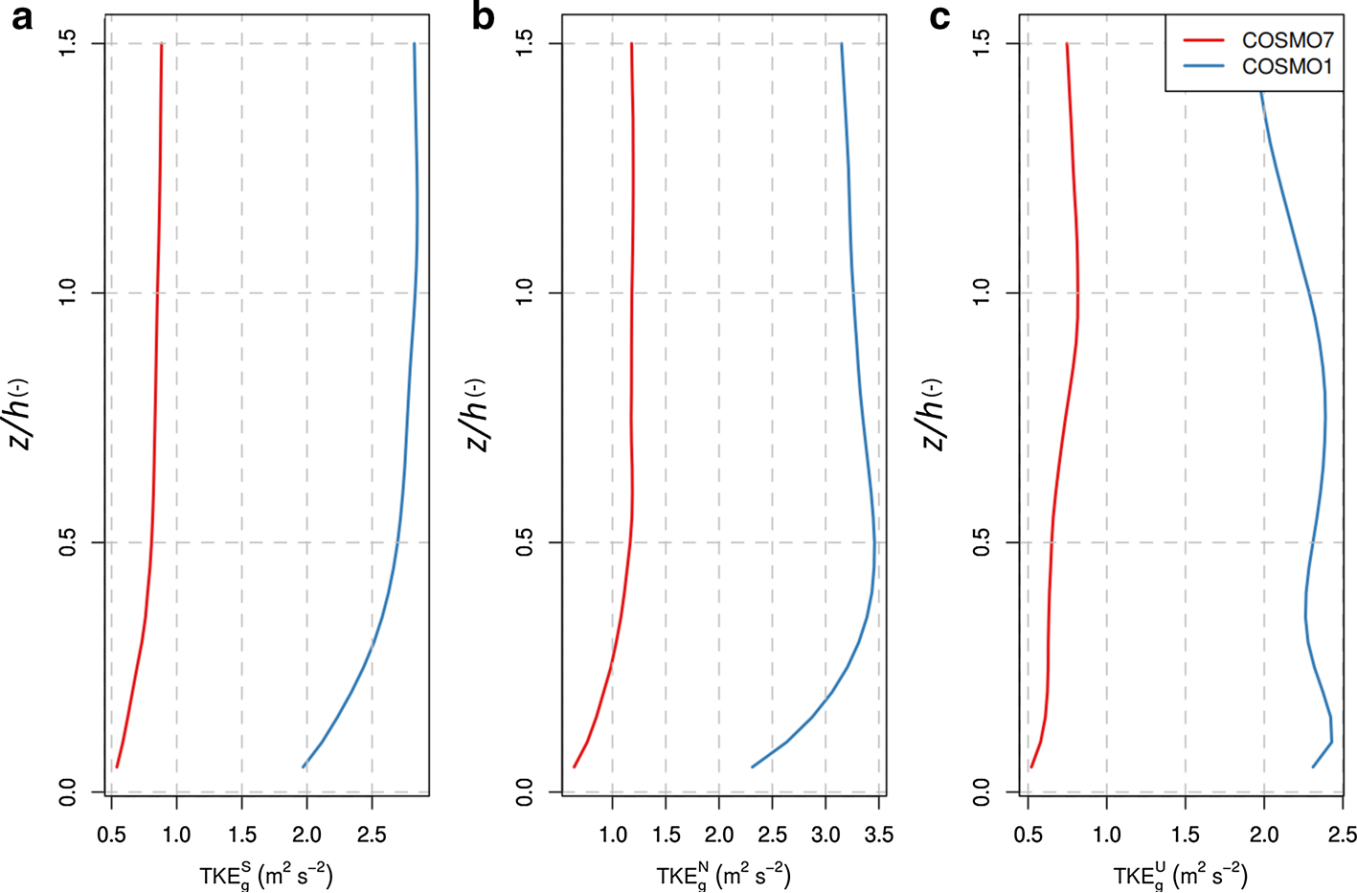
Comparisons of annual mean vertical profiles of grid-resolved TKE between COSMO-1 and COSMO-7: **a** stable, **b** neutral, and **c** unstable conditions ($\hbox {TKE}^S_g$, $\hbox {TKE}^N_g$, and $\hbox {TKE}^U_g$ respectively). The vertical axis is normalized by the ABL height

As described in Sect. 2.2, FLEXPART’s turbulence scheme provides estimates for the variations of the winds for the three different stability classes (neutral, unstable, and stable). The separation to the different stability classes is based on the value of the dimensionless parameter *h*/*L*. In order to compare the turbulent intensity in FLEXPART-COSMO with that in COSMO, we computed TKE, *e*, which is defined as half of the sum of the variances of the wind components:

12 $$\begin{aligned} e= & {} \frac{1}{2}\left( \overline{u^{'}u^{'}}+\overline{v^{'}v^{'}} +\overline{w^{'}w^{'}}\right) , \end{aligned}$$

13 $$\begin{aligned} \sigma _{u}^{2}= & {} \overline{u^{'}u^{'} }=\frac{1}{T}\int _{0}^{T}(u(t)-{\overline{u}})^{2}\mathrm{{d}}t= \frac{1}{\int _{{{\textbf {S}}}}\mathrm{{d}}x\mathrm{{d}}y}\int _{{{\textbf {S}}}}(u(x,y)-{\overline{u}})^{2}\mathrm{{d}}x \mathrm{{d}}y, \end{aligned}$$

where *T* represents the period and ${\mathbf {S}}$ the domain over which the wind components are averaged. Equation 13 results from the definition of the variance and the ergodic theorem and holds for all three components of the wind. Exploiting Eqs. 12 and 13 we calculated the diagnosed/parameterized TKE for FLEXPART-COSMO and compared it to the prognostic TKE in COSMO, since our notion was that FLEXPART’s turbulence scheme overestimates TKE. We additionally approximated the grid resolved TKE in COSMO, $\hbox {TKE}_g$, from horizontal variability of COSMO’s wind fields within a limited area of 30 $\times$ 30 $\mathrm {km^2}$ around the tall tower site BRM. The size of the area is a compromise between relatively homogeneous terrain and including a sufficiently large number of grid cells. $\hbox {TKE}_g$ can be seen as the part of the total turbulence in the atmosphere that the NWP model can resolve by itself without the help of a subgrid-scale parametrized scheme. In principle, the sum of the prognostic/diagnostic TKE and resolved small-scale variability (grid resolved turbulence, $\hbox {TKE}_g$) should be the total amount of turbulence present in the model (Honnert et al. 2011). However, we acknowledge that increases of $\hbox {TKE}_g$ at finer resolution may also arise from a more adequate representation of mesoscale flow features (especially in complex terrain) and are not solely of turbulent nature. Consequently, larger $\hbox {TKE}_g$ at finer resolution should also lead to enhanced dispersion. For the area around BRM we calculated the standard deviation of the topography (similar to $\hbox {TKE}_g$) to be a factor of four larger for COSMO-1 as compared to COSMO-7. Vertical profiles of COSMO’s prognostic TKE and grid-resolved $\hbox {TKE}_g$ inside the ABL were extracted from COSMO-1 and COSMO-7 archived fields for the whole year 2016 with a temporal resolution of the data of 1 h. Similarly, these COSMO fields were used to diagnose TKE with FLEXPART’s Hanna (1982) scheme. The annual mean vertical profiles are displayed in Figs. 2 and 3 .

Figure 2 shows that for neutral and unstable conditions, prognostic TKE is always larger in COSMO-7 than in COSMO-1. Conceptually, this agrees with the idea that at high resolution and when ABL heights reach values over 1 km during unstable conditions, a large part of the turbulence spectrum is already resolved by COSMO-1 grid-scale winds and doesn’t need to be parameterized. COSMO’s turbulence scheme seems to mimic this resolution dependency. $\hbox {TKE}_g$, in contrast, is 3–5 times higher in COSMO-1 than in COSMO-7 for all stability classes (Fig. 3). This is a clear indicator of the difference between the resolved turbulence in the two models. It is in line with the theory behind the grey zone of turbulence (Honnert et al. 2020), stating that with the increasing resolution the grid-resolved TKE should be increasing. Because $\hbox {TKE}_g$ is several times larger in COSMO-1 than in COSMO-7, total TKE (resolved plus subgrid) is considerably larger in COSMO-1 than in COSMO-7. Part of this difference may arise from the differences in the resolved topography between the two models and the degree to which terrain-induced flow variability is covered.

TKE as parameterized in FLEXPART-COSMO corresponds to the dashed lines in Fig. 2. It is consistently larger than the prognostic TKE in COSMO during neutral and especially unstable conditions. This overestimation is more pronounced at higher resolution. FLEXPART’s Hanna (1982) scheme does not consider any dependency of unresolved turbulence on grid resolution and, as a consequence, profiles remain similar for different model resolution. In contrast to the neutral and unstable cases, COSMO TKE is larger than FLEXPART TKE during stable situations. The difficulty of COSMO to represent stable boundary layers is well known and has been described (Brunner et al. 2015; Westerhuis et al. 2020).

### Modified Equations of Wind Variances

To align FLEXPART’s diagnosed TKE with COSMO’s prognostic TKE, at high resolution, we introduce a modification of the Hanna (1982) turbulence scheme for unstable and neutral conditions. Recently, Verreyken et al. (2019) developed a turbulence scheme for FLEXPART-AROME, which directly utilizes TKE from the Eulerian model for the representation of turbulence in FLEXPART. The scheme was further applied by Cornwell et al. (2021) in a large-eddy simulation (LES) configuration, which also highlights the importance of balancing grid-resolved and parameterized turbulence. However, a previous study with another LPDM coupled to COSMO in complex terrain suggested that a similarity approach for the LPDM’s turbulence description, like Hanna (1982), may be more accurate than the direct use of COSMO TKE (Szintai et al. 2010). They attributed this problem to the patchiness found in individual TKE profiles and distributions, which may degrade dispersion simulations.

Our new turbulence scheme was implemented within the framework of similarity theory through a new set of equations of the variations of the winds that were obtained by fitting a nonlinear least-square (NLS) regression model to the hourly available COSMO-1 TKE values, using the four main turbulence parameters [friction velocity $(u_{*})$, convective velocity scale $(w_{*})$, Obhukov Length (*L*), ABL height (*h*)] and height above ground (*z*) as predictor variables. Obtaining a diagnostic TKE in FLEXPART as a continuous function of height has the advantage that vertical gradients of the vertical variations of the wind (as required by the drift correction in Eq. 5) are analytically available and don’t have to be expressed by finite difference approximations.

#### Neutral Conditions

Starting from the neutral case (defined in FLEXPART as $\frac{h}{|L|} < 1$) and using Hanna (1982)’s functional relationship for the variations of the winds, diagnostic TKE can be written as:

14 $$\begin{aligned} e =\alpha u^{2}_{*}\exp {\left( \zeta \frac{fz}{u_{*}}\right) } +\beta u_{*}^{2}\exp {\left( \kappa \frac{fz}{u_{*}}\right) }, \end{aligned}$$

where $\alpha , \zeta , \beta$ and $\kappa$ are parameters to be estimated by a regression model. The same Greek letters are used for the parameters of the TKE equations to be determined by the regression model, both in neutral and unstable conditions, but they exhibit different values across the different stability regimes. The values of these parameters for the Hanna (1982) equation are $\alpha =2$, $\zeta =-6$, $\beta =1.69$, and $\kappa = -4$. In order to estimate the parameters best describing the COSMO TKE profiles, we fit the above function to the hourly COSMO TKE values, $y_1,y_2,\ldots ,y_n$, using a NLS regression model (Bates and Watts 2008), which minimizes the residual sum of squares (Eq. 15) with respect to the vector of the parameters (**p**):

15 $$\begin{aligned} RSS({{\textbf {p}}})=\sum _{i=1}^{n}(y_{i}-g(x_{i};{{\textbf {p}}}))^{2}, \end{aligned}$$

where $x_i$ are the independent variables (here $u_*$ and *z*) of the function *g* (here Eq. 14), and *i* ranges over both height above ground and time. We used hourly TKE profiles for the whole year 2016. There is a set of additional constraints in the nonlinear regression, imposing conditions to be satisfied by each term of the diagnostic TKE formulas. The constraints governing the neutral case are:

- $e= \frac{1}{2}(\sigma _{u}^{2}+\sigma _{v}^{2}+\sigma _{w}^{2})$; from which follows that each term of the formulas used in the nonlinear regression to parameterize TKE must be positive.
- Each term of the equations for the variations of the winds should have the dimension of energy.
- Relative contributions to diagnostic TKE by each of the terms should be similar, as in Hanna (1982), in order to represent the attribution to the different flow directions, also considering that the different turbulence components follow different vertical profiles as suggested by Hanna (1982).
- Functional relationships for the variations of the winds must be continuous and differentiable functions of the height *z*.

To determine whether there is any other functional relationship hidden between COSMO TKE and the predictor variables $(h,L,u_{*},z)$, a regression with a generalized additive model (GAM) (Wood 2006) was utilized, where the COSMO TKE values were used as a response and where different combinations of the predictor variables leading to dimensionless quantities or quantities with dimension of energy were used as predictors $(u_{*}^{2}, \frac{z}{L}, \frac{z}{h}, \frac{h}{L}, \frac{fz}{u_{*}})$. The derived GAM model (Online Resource S1) revealed that there is no strong relationship for the predictors $\frac{z}{L},\frac{h}{L}$ but there is a relationship for normalized height, $\frac{z}{h}$, which is not included in the original Hanna (1982) scheme (Eq. 7). To accommodate this effect, we added a third term in Eq. 14.

Furthermore, to reduce the number of parameters in the equation and since the shape of COSMO-1 TKE and FLEXPART-COSMO-1 TKE in Fig. 2 are similar, we kept only the parameters in front of the two exponential terms in Eq. 14 but fixed the values of $\zeta$ and $\kappa$ to those used by Hanna (1982). The new diagnostic TKE equation that was used in the nonlinear regression model is then given by:

16 $$\begin{aligned} e =\alpha u^{2}_{*}\exp {\left( -6 \frac{fz}{u_{*}}\right) } +\beta u_{*}^{2}\exp {\left( -4 \frac{fz}{u_{*}}\right) }+ \gamma u_{*}^{2}\exp {\left( \delta \frac{z}{h}\right) }. \end{aligned}$$

#### Unstable Conditions

For the unstable case we followed a slightly different approach for the determination of the formulas for the variations of the wind. Browsing through individual TKE profiles of COSMO-1 during unstable conditions, we observed that they do not all follow a common shape, but that there exist different dominant patterns. Since these general shapes differ substantially from each other, different formulas should be used during unstable conditions. In order to develop a robust criterion to distinguish them, we applied a hierarchical clustering method (Murtagh and Contreras 2012) with a cosine similarity measure to separate COSMO TKE profiles for unstable cases within the whole year 2016 into different categories. The cosine similarity is based on the dot product between two vectors ${{\textbf {a}}}$ and ${{\textbf {b}}}$, where the cosine of the angle between two vectors can be written as fraction of their product divided by their magnitude:

17 $$\begin{aligned} cos(\theta ) = \frac{{{\textbf {a}}}{{\textbf {b}}}}{|{{\textbf {a}}}||{{\textbf {b}}}|}. \end{aligned}$$

In our case, each vector represents an individual COSMO TKE profile at a given time, comprised of 20 elements which correspond to different normalized height levels. The resulting similarity (angle between the vectors) ranges from $-1$ meaning exactly opposite, to 1 meaning exactly the same, with 0 indicating orthogonality of the vectors, whereas in-between values indicate intermediate similarity or dissimilarity. Thus, we expect that similarly shaped profiles will have higher values of cosine similarity between them irrespective of their TKE magnitude and the clustering method should be able to distinguish them.

The clustering suggested that the profiles can be grouped into two main clusters and that further division in sub-clusters does not reveal additional general profile shapes. The mean TKE profiles of the two dominant clusters can be described as follows: cluster-1 has a relatively flat parabolic shape, whereas cluster-2 has an exponential shape (Online Resource S2). Cluster-1 accounts for about 30% of all unstable cases, cluster-2 for about 70%. The magnitude of TKE was found to be considerably higher for cluster-2 than for cluster-1.

**Fig. 4:**
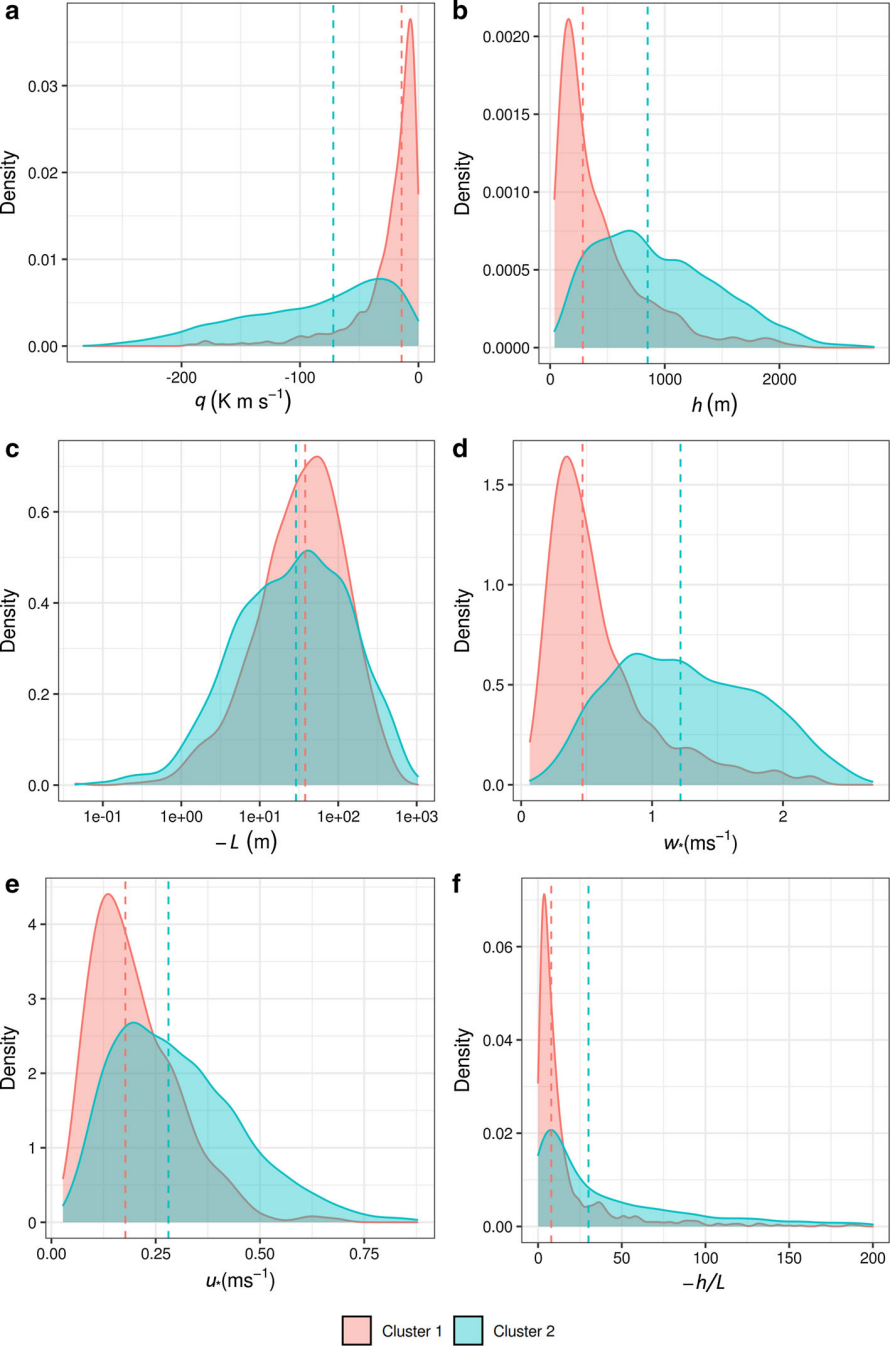
Probability density distributions of **a** surface sensible heat flux, **b** boundary-layer height, **c** Obhukov length, **d** convective velocity scale, **e** friction velocity, and **f** relative Obhukov length for the two clusters during unstable conditions. The dashed vertical lines correspond to the mean value of the distributions

Since the stability categorization based on the clustering approach is not an available parameter during a FLEXPART run, an alternative parameter needs to be selected for the purpose of dividing the unstable cases into sub-categories. For the two different categories of COSMO TKE profiles, we compared the distributions of the four main turbulence parameters used as predictors of COSMO TKE, ($u_{*}$, $w_{*}$, *L*, *h*), the surface sensible heat flux, *q*, and the relative Obhukov length, $-h/L$, to spot potential differences. Cluster-1 generally exhibits lower values of $w_{*}$ than cluster-2. The exponentially shaped profiles of cluster-2 reflect fully or nearly fully developed boundary layers as can be seen in Fig. 4, where the average ABL height for cluster-2 is significantly larger than for cluster-1. Cluster-1 includes more shallow, probably still developing convective boundary layers. Surface heat fluxes are also lower (Fig. 4) in cluster-1. These convective boundary layers (CBLs) may be early morning developing boundary layers or cases of CBLs with clouds or after cloudy conditions. The eddies of such shallow CBLs should be considerably smaller when compared to the mesh of the NWP model and, hence, would be unresolved by the grid-scale wind and instead should be covered by the turbulence parametrized. On the other hand, in the fully developed CBLs, a considerable part of the big thermals may be resolved by grid-scale winds of the high-resolution model, leaving only the medium- and small-sized eddies to be accounted for by the parametrized scheme. The latter may be the main reason for the difference between the TKE shapes of cluster-1 and cluster-2.

Since $w_{*}$ contains the information about the height of the ABL and the surface heat flux and is the parameter with the least overlap between the density distributions of cluster-1 and cluster-2 (Fig. 4), we decided to use it for the separation of the unstable cases in FLEXPART. We selected a cut-off value of 0.45 $\mathrm {m~s^{-1}}$, which is only a little higher than the mean value of cluster-1 but includes about 60$\%$ of all cluster-1 cases. To arrive at this cut-off value for the categorisation by $w_{*}$, we determined the intersection point between the $w_{*}$ histograms for the two clusters. At this point, the difference between $w_{*}$ cases from cluster-1 and cluster-2 included in the first category is maximized. There is always an overlap between cluster-1 and cluster-2, as can be seen in Fig. 4, but with the selected cut-off value of $w_{*}$ the overlap does not significantly influence the average TKE profile of each different cluster.

In a next step, the diagnostic TKE equations for both sub-categories were derived using the same principles as in the neutral case. For both sub-categories, the derivation started using the Eqs. 8 and 9 trying at the same time to satisfy the same constrains applied during neutral conditions with the addition of one more:

- Equations for the two clusters should diagnose similar TKE values for the critical value of $w_{*}=0.45$.

Deriving formulas based on Eqs. 8 and 9 , which at the same time respect the constrains described above, proved very challenging. When enforcing these constraints in the nonlinear regression, the solutions for the parameters led to poorly fitted models. A modification of the equations was therefore considered to obtain improved models. Again, GAM models were utilized for the two clusters in order to identify those predictors that explain most of the COSMO TKE variability (Online Resources S3 and S4), and quantify the amount of variability that can be explained from these predictor variables $u_{*}$, $w_{*}$, *z*/*h*, *h*/*L*, *z*/*L* . The same predictor variables used in Hanna (1982) formulas were found to be the ones best explaining COSMO TKE. Thus, we used them as predictors to fit the COSMO TKE values while modifying the functional relationships to reproduce the COSMO TKE profiles.

For the cluster-1 and for the horizontal components of the variations of the winds, the same formula as in Hanna (1982) was used (Eq. 8). This term lacks any dependency on height, hence contributing a constant amount to TKE throughout the ABL. For the vertical term and since we need a parabolic profile shape (Online Resource S2), we fixed all the dimensionless independent variables, ($\frac{z}{h}$), of the equation in the power of 2 instead of $\frac{2}{3}$ as in the original Hanna (1982) equation. The resulting form of the diagnostic TKE equation is given by:

18 $$\begin{aligned} e = \alpha u_{*}^2\left( 12+\frac{h}{|2L|}\right) ^{2/3}+\beta \left( 1.2w_{*}^2\left( 1-\gamma \frac{z}{h}\right) ^{2}+\delta \left( 1.81-\lambda \frac{z}{h}\right) ^{2} u_{*}^{2}\right) . \end{aligned}$$

For cluster-2, and since we have to fit an exponential profile, we kept the same term for the variations of the winds for the horizontal components as in Hanna (1982) for the same reason as in cluster-1, and we added two more exponential terms based on $u_{*}$ and $w_{*}$, respectively, resulting in:

19 $$\begin{aligned} e = \alpha u_{*}^{2}\left( 12+\frac{h}{|2L|}\right) ^{2/3}+\beta w_{*}^{2}\exp {\left( \gamma \frac{z}{h}\right) }+\delta u_{*}^{2}\exp {\left( \lambda \frac{z}{h}\right) }. \end{aligned}$$

#### Stable Conditions

Finally, the Hanna (1982) relationships for stable cases were left untouched. On the one hand, Hanna (1982) diagnoses smaller TKE values in these situations than COSMO predicts, while COSMO is known to overestimate mixing in stable situations (Brunner et al. 2015; Westerhuis et al. 2020). On the other hand, boundary-layer heights are usually shallow during stable conditions and eddy sizes can be assumed to be smaller than COSMO grid sizes. Thus, these turbulence structures fully remain a subgrid process and a modification due to mesh size seems unnecessary.

### Lagrangian Time Scales

The Langevin equation, Eq. 5, predicting the turbulent wind fluctuations, needs functions for the variations of the winds and the Lagrangian time scales of the auto-correlation of the velocities in three dimensions. The Lagrangian time scales, $\tau _{L_{j}}$, are estimates of the time for which the stochastic deviation of the wind has a memory of the stochastic wind increment at a previous time:

20 $$\begin{aligned} \tau _{L_{j}}= & {} \int _{0}^{\infty }R_{j}^{L}(\tau )\mathrm{{d}}\tau , \text {and} \end{aligned}$$

21 $$\begin{aligned} R_{j}^{L}(\tau )= & {} \frac{\overline{u^{'i}_{j}(t)u^{'i}_{j}(t+\tau )}}{\sigma _{j}^{2}}, \end{aligned}$$

where $R_{j}^{L}$ is the Lagrangian auto-correlation function for direction *j*, taking values from -1 to 1, and $\tau$ the time lag. $u^{'i}_{j}(t) = u^{i}_{j}(t)-\overline{u^{i}_{j}(t)}$ is the velocity fluctuation of particle *i* at time *t*, and the overbar is the average over all particles. The dispersion of particles moving in a turbulent flow is an evolutionary process (time dependent), and it is formulated as (Csanady 1973):

22 $$\begin{aligned} \overline{x_{j}^{'2}(t)}=2\sigma ^{2}_{j}\int _{0}^{t}\int _{0}^{t'} R_{j}^{L}(\tau )\mathrm{{d}}\tau \mathrm{{d}}t'. \end{aligned}$$

Equation 22 has two analytical limits for the near and far field dispersion respectively,

23 $$\begin{aligned} \overline{x_{j}^{'2}(t)} = {\left\{ \begin{array}{ll} \sigma _{j}^{2}t^{2} &{} t<< \tau _{L_{j}} , \\ 2\sigma _{j}^{2}\tau _{L_{j}}t &{} t>> \tau _{L_{j}} \end{array}\right. }. \end{aligned}$$

From Eq. 23 we see that the far field dispersion is analogous to the variations of the winds and the Lagrangrian time scales. The larger the Lagrangian time scales, the faster the dispersion at the same values of $\sigma _j$. This is intuitive since the higher the Lagrangian time scales the higher the persistence of the turbulent fluctuations to retain their previous values. Large thermals with big spatial dimensions are expected to exhibit higher values of Lagrangian time scales when compared to small eddies. Since big thermals are partly resolved by the high-resolution NWP models, the remaining smaller eddies, which need to be taken care of by the turbulence scheme, should have smaller Lagrangian time scales.

No equations based on fundamental physical laws exist for the Lagrangian time scales. The functional relationships used in most LPDMs are based on Lagrangian wind measurement experiments or on relating the Lagrangian time scales to the more commonly observed Eulerian time scales (Ryall and Maryon 1998; Dosio et al. 2005). The equations used in Hanna (1982) for the Lagrangian time scales are inversely proportional to the variations of the winds, $\sigma _{j}$. These relationships were built for the peak of the turbulence energy spectrum (Ryall and Maryon 1998) and are representative for the variations of the winds over the full turbulence energy spectrum. If we apply these Lagrangian time scale equations together with our newly derived equations for the variations of the winds, we obtain very large values for $\tau _{L_{j}}$, up to 5–10 times larger than with Hanna (1982), which is physically unrealistic if we assume that the big thermals are partly resolved and the remaining structures to be parameterized have dimensions smaller than the grid resolution, which should translate into smaller Lagrangian time scales.

Lagrangian time scales may also be calculated from TKE dissipation, $\epsilon$, by:

24 $$\begin{aligned} \tau _{L_{j}} = \frac{2\sigma _{j}^2}{C_{0}\epsilon }, \end{aligned}$$

where $C_{0}$ is a universal constant with values in the literature ranging from 2 to 10 (Rodean 1996). We considered different ways of calculating TKE dissipation and subsequently Lagrangian time scales following suggestions in the literature (Ryall and Maryon 1998; Rodean 1996; Goger et al. 2018; Degrazia et al. 1998). The resulting values of Lagrangian time scales proved to be unfit for the use in FLEXPART either due to very high-simulated values or due to vertical profiles that were not compatible with the well-mixed criterion and the currently implemented simplification of the drift term in the Langevin equation (see Eq. 5). In the end, we chose to stay with the newly derived wind variations and the inverse relation between $\sigma _j$ and $\tau _{L_{j}}$ as given by Hanna (1982), but we introduced a scaling factor to tune resulting time scales to values more consistent with the previous Hanna (1982) estimates. The resulting values should be slightly lower than in Hanna (1982), since the largest eddies are not represented by the new scheme anymore. To estimate an optimal value of the scaling factor for unstable conditions, several test-runs varying its value and exploring different boundary-layer parameters ($w_{*},L,h,u_{*}$) were performed. For neutral conditions the time scales were not adjusted from the Hanna (1982) formulation.

## Results and Discussion

### Final Set of Equations for the Variations of the Winds

The analysis described in Sect. 2.5 leads to the following set of equations for the variations of the winds for neutral and unstable conditions (Eqs. 25–30). The equations for the vertical derivatives of the variations of the winds can be found in the supplement (Eqs. 1–3), while for the Lagrangian time scales we utilized the same equations as in Stohl et al. (2005). The equations for the three components, the two horizontal and the one vertical component, of the variations of the winds were extracted from the derived diagnostic TKE equations in a similar manner as in Hanna (1982). The variables with Greek letters correspond to parameters determined by the nonlinear regression model.

- *Neutral Conditions:*
  25 $$\begin{aligned} \sigma _{u}= & {} \sqrt{2\alpha } u_{*}\exp {\left( \frac{-3fz}{u_{*}}\right) }, \text {and} \end{aligned}$$
  26 $$\begin{aligned} \sigma _{v}= & {} \sigma _{w}= \sqrt{u_{*}^{2}\beta \exp {\left( \frac{-4fz}{u_{*}}\right) +u_{*}^{2}\gamma \exp {\left( \frac{\delta z}{h}\right) }}}. \end{aligned}$$
- *Unstable conditions, cluster-1 *($w_* < 0.45$):
  27 $$\begin{aligned} \sigma _{u}= & {} \sigma _{v}=u_{*}\sqrt{\alpha } \left( 12+\frac{h}{2|L|}\right) ^{\frac{1}{3}}, \text {and} \end{aligned}$$
  28 $$\begin{aligned} \sigma _{w}= & {} \sqrt{\beta \left( 1.2w_{*}^{2}\left( 1+\frac{\gamma z}{h}\right) ^{2} +\delta \left( 1.81-\lambda \frac{z}{h}\right) ^{2}u_{*}^{2} \right) }. \end{aligned}$$
- *Unstable conditions, cluster 2* ($w_* \ge 0.45$):
  29 $$\begin{aligned} \sigma _{u}= & {} \sigma _{v}=u_{*}\sqrt{\alpha }\left( 12+\frac{h}{2|L|}\right) ^{\frac{1}{3}} , \text {and} \end{aligned}$$
  30 $$\begin{aligned} \sigma _{w}= & {} \sqrt{\beta w_{*}^{2}\exp {\left( \gamma \frac{z}{h}\right) }+\delta u_{*}^{2}\exp {\left( \lambda \frac{z}{h}\right) }}. \end{aligned}$$

To determine the individual tuning parameters, Eqs. 16, 18, and 19 for neutral and unstable conditions, respectively, were fitted to one year (2016) of hourly COSMO TKE profiles averaged over an area of $30 \times 30 \hbox {km}^2$ centred at the tall tower location of BRM. Table 1 summarizes the corresponding values of the parameters determined by the nonlinear regression model.

**Table 1:** Values of the parameters of the formulas of the variations of the winds for the neutral and unstable case

Variable	Neutral	Unstable cluster-1	Unstable cluster-2
$\alpha$	1	0.27	0.11
$\beta$	1	0.33	0.16
$\gamma$	1.91	$-$ 0.16	$-$ 3.6
$\delta$	$-4.38$	1.18	1.94
$\lambda$	–	2.28	$-$ 2.69

**Fig. 5:**
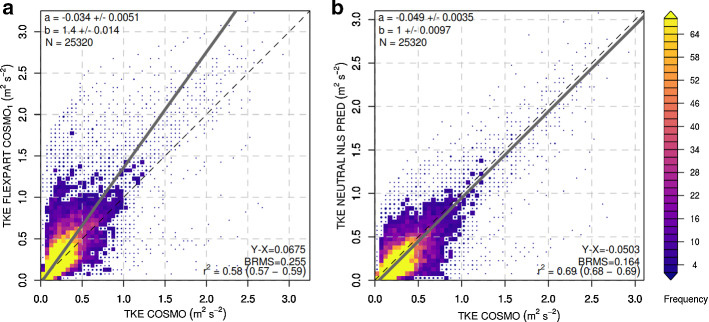
Density scatter plots of the TKE values between the COSMO predictions (*x* axis) and FLEXPART diagnosed TKE (*y*-axis) for **a** the Hanna (1982) scheme and **b** the new scheme proposed in this study, both during neutral stability. Colours represent the number of data points (frequency) in a given grid area. Regression slopes were calculated through orthogonal least-square regression. The black values in the plot correspond to: (a) is the regression intercept; (b) is the regression slope. *N*, number of data points; $Y-X$, mean bias; BRMS, bias-corrected root mean square error; $r^{2}$, coefficient of determination and its uncertainty range

Figure 5 assesses the relationship between FLEXPART diagnosed TKE (both Hanna (1982) and our new scheme) and COSMO predicted TKE for different levels in the ABL during neutral conditions. When we compare the two FLEXPART parameterizations, it is clear that the new diagnostic TKE formula (Eq. 16) is (1) better correlated with COSMO TKE and (2) yields more similar TKE levels. The Hanna (1982) equations for the neutral case overestimates TKE when compared with COSMO, while the new equation produces equal amounts on average. By comparing the statistics presented in Online Resource S5 and Fig. 5, we can also see that the performance of the nonlinear regression model is only slightly inferior to the performance of the non-parametric GAM, which can be seen as an upper bound of the predictability considering the selected predictor variables.

**Fig. 6:**
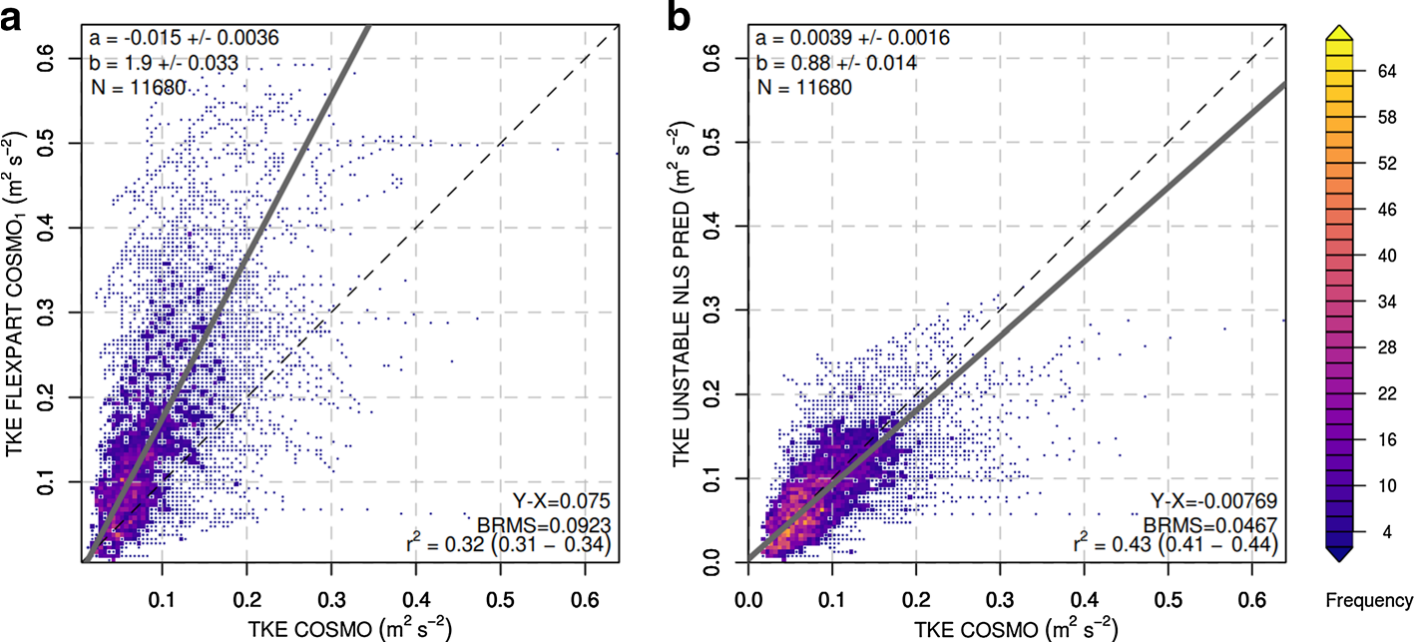
Density scatter plots between COSMO TKE (*x* axis) and FLEXPART diagnosed TKE (*y*-axis) for **a** the Hanna (1982) scheme and **b** the new scheme proposed in this study, both during cluster-1 unstable cases. Colours represent the number of data points (frequency) in a given grid area. Regression slopes were calculated through orthogonal least-square regression

For unstable cluster-1 conditions, the new scheme outperforms Hanna (1982) even more clearly (Fig. 6). Hanna (1982) overpredicts TKE almost by a factor of two (regression slope of 1.9), whereas the new scheme brings diagnosed TKE values in much closer agreement with prognostic TKE in COSMO.

**Fig. 7:**
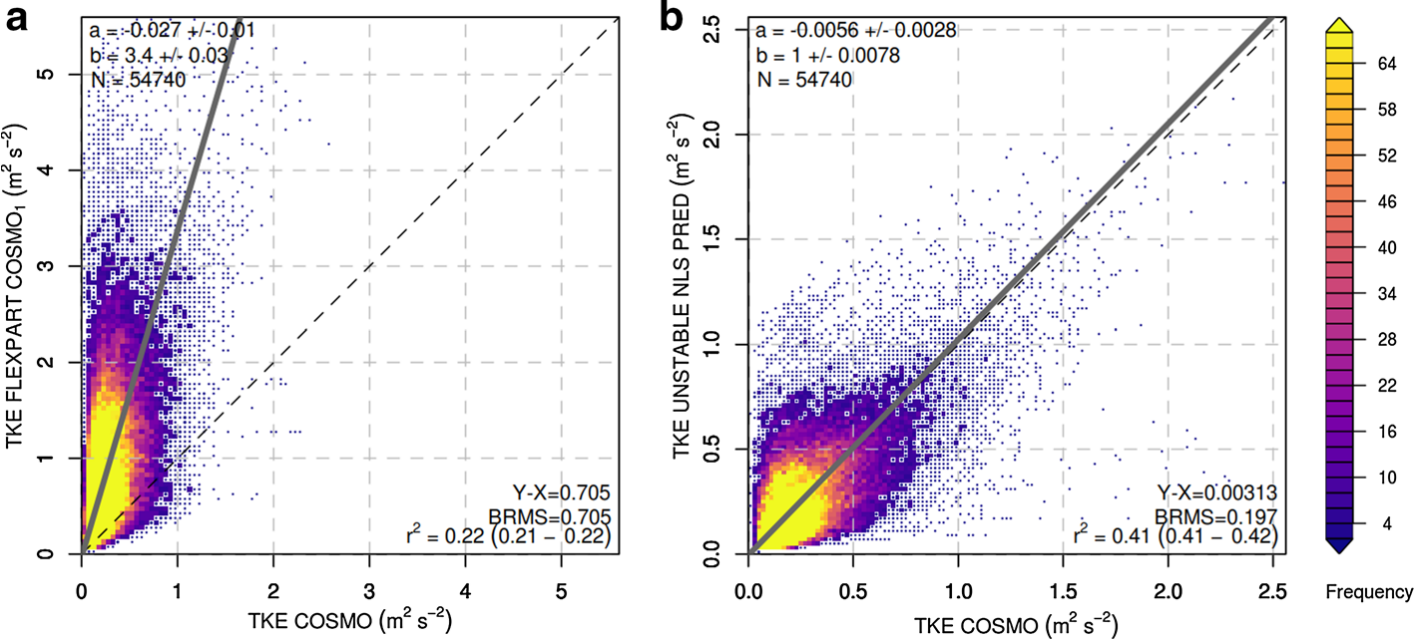
Density scatter plots between COSMO TKE (*x* axis) and FLEXPART diagnosed TKE (*y*-axis) for **a** the Hanna (1982) scheme and **b** the new scheme proposed in this study, both during cluster-2 unstable cases. Colours represent the number of data points (frequency) in a given grid area. Regression slopes were calculated through orthogonal least-square regression

For unstable cluster-2, the largest discrepancy between Hanna (1982) TKE and COSMO TKE is visible, with COSMO TKE mostly remaining at values below 1 $\mathrm {m^2~s^{-2}}$, whereas Hanna (1982) TKE reaches values larger 3 $\mathrm {m^2~s^{-2}}$ (Fig. 7). The new scheme brings the general levels of TKE into much better agreement with COSMO TKE. However, from the large remaining scatter and comparatively low coefficient of determination it is clear that a simple parametrization of COSMO TKE becomes increasingly challenging under more and more unstable conditions.

For both unstable categories, the variability of COSMO TKE explained by the nonlinear regression remained considerably smaller than for the neutral case. GAM estimates for the unstable cases were able to explain a larger fraction of variability than the nonlinear regression models, but were biased low for large COSMO TKE values (Online Resources S6 and S7).

### Universality of the New Turbulence Kinetic Energy-based Scheme

The tuning of FLEXPART’s turbulence scheme to match the TKE produced by COSMO was implemented for the BRM region on the Swiss Plateau. An obvious question is if the new formulas for neutral and unstable conditions also hold for other locations, especially with respect to different degrees of topographic complexity. For that reason, we repeated the TKE analysis for different locations within the COSMO-1 domain: one area close to Saint Gallen in eastern Switzerland with slightly more complex topography compared to BRM, and areas in flat terrain (close to Munich, Germany, eastern France, and the Po Valley in North Italy). At all locations, the new scheme as derived from the regression at BRM was applied and compared to COSMO TKE. From this comparison (Online Resources S8–S19) it is apparent that the new turbulence scheme outperforms the Hanna (1982) parametrization at all sites in terms of reproducing COSMO’s TKE. As for BRM, the biggest differences can be seen during unstable conditions and for cluster-2. Hanna (1982) TKE values are three to four times higher when compared to COSMO for this cluster, while the tuned turbulence scheme produces similar values as COSMO. It would be presumptuous to state that the new equations for the variations of the winds show universal validity, but at least we can claim that they provide a very good fit for the COSMO-1 domain and the operational setup used by MeteoSwiss.

### Turbulent Mixing and Well-Mixed Tests

To assess the general behaviour of the newly derived equations, we set up idealized mixing experiments, where particles were released near the surface at a single point in time and the subsequent vertical position in the ABL was monitored. For these simulations, only vertical mixing by parameterized turbulence was permitted, by setting mean winds and horizontal wind variations to zero. Other meteorological conditions (surface fluxes, temperature profile, ABL height, etc.) were kept constant over time. In this configuration, model particles remain within the ABL due to the implemented reflection at the ABL top. Two aspects of the turbulence parametrization are addressed by these tests. First, the time scales of turbulent vertical mixing for different configurations can be analyzed in isolation without any influence from grid-resolved transport. Second, the compliance of the turbulence scheme with the well-mixed criterion can be verified. An initially well-mixed particle distribution within the ABL should remain well-mixed also after a long integration time. Here, we assess this particle distribution by evaluating the number of particles in separate layers in the ABL divided by total number of particles in the simulation and multiplied by the number of layers, $\tau$. In the absence of density gradients, $\tau$ should take a value of 1 everywhere in the ABL. In order to account for density gradients, we further divide $\tau$ by the air density, $\rho$, so that $\tau /\rho$ should take a constant value throughout the ABL.

**Fig. 8:**
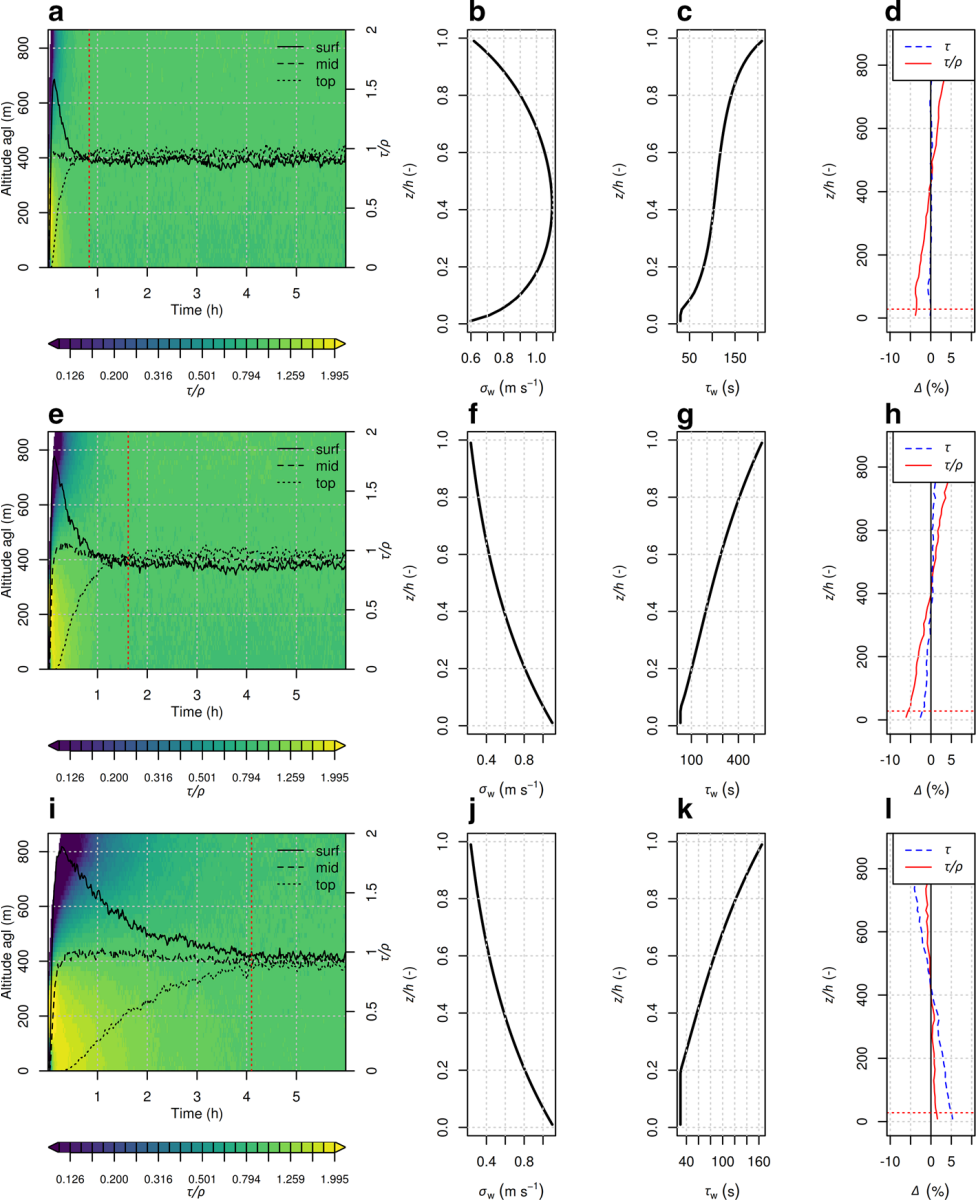
Sub-panels in the leftmost column show the vertical concentration profile as a function of time (*x* axis) and height (*y* axis). The vertical concentration corresponds to normalized particle distribution divided by air density, $\tau /\rho$ . The black lines correspond to values of $\tau /\rho$ at the bottom, mid, and top of the ABL. Vertical red dashed lines give the time when the concentration at top approaches the expected final concentration within a $\pm 5\%$ margin. Sub-panels in the second and third column show vertical profiles of $\sigma _{w}$ and $\tau _{L_{w}}$, and in the fourth column sub-panels show the result of a well-mixed test for the same meteorological conditions. The first row corresponds to simulations with Hanna (1982) turbulence scheme, the second row to simulations with the new turbulence scheme but with Hanna (1982) estimates for the Lagrangian time scales, and the third row to TKEts simulations. The simulations were initialized for the COSMO grid cell located at BRM and at 1200 UTC 12/5/2016

Figure 8 shows the results of three different mixing experiments, one utilizing the Hanna (1982) scheme, one with the newly derived equations for the variations of the winds but with Hanna (1982) relationships for the Lagrangian time scales, and one with the newly derived equations for the variations of the winds and Lagrangian time scales reduced by $\sim$70 %. We will refer to the last configuration as (TKEts) in the following. These experiments correspond to a typical cluster-2 unstable case with a fully developed ABL ($w_{*}=1.56\,\mathrm {m~s^{-1}}$, $u_{*}=0.35\,\mathrm {m~s^{-1}}$, $L = -28\,\mathrm {m}$, $h=867\,\mathrm {m}$).

It can be seen that the Hanna (1982) turbulence scheme (Fig. 8a) leads to very rapid mixing of the tracer throughout the ABL. When we utilize the newly derived relationships for the variations of the winds (Fig. 8e), the mixing becomes somewhat slower, but not sufficiently slow to have a significant impact on the time when concentrations at the ABL top become similar to those in the lower ABL. This is due to the fact that the Lagrangian time scales (Fig. 8g) exhibit unrealistically large values when we use the new equations for the variations of the winds as described in Sect. 2. Large Lagrangian time scales mean that particles receiving a large vertical velocity component near the ground will preserve this velocity over a long time due to the long memory and subsequently will rise quickly throughout the ABL. When we reduce the Lagrangian time scales by 70% (Fig. 8i) the mixing becomes considerably slower, something which is in line with Eq. 23. Thus, we chose a simple down-scaling of $\tau _{L_{w}}$ as given by Hanna (1982). The resulting profile has the advantage of monotonously increasing values for $\tau _{L_{w}}$ with height, which facilitates well-mixedness in our tests. Even smaller values would result in slower mixing but also increased stratification by the end of the simulation. It should be stressed again that the mixing in Fig. 8 only represents the mixing by unresolved turbulence. In the complete model, grid-resolved motion will add to the mixing, and the time until vertical concentration gradients disappear will again be shorter.

We performed some further tests to examine whether the TKEts configuration satisfies the well-mixed criterion (Thomson 1987). The setup was the same but the tracer was well-mixed in the ABL already at the beginning of the simulation and not emitted near the surface. We can see that even the default turbulence scheme of FLEXPART (Hanna 1982) did not satisfy the well-mixed criterion, but there is a prominent vertical gradient which leads to some accumulation of particles at the top of the ABL and de-accumulation at the surface (Fig. 8d).The blue dashed line corresponds to the residence time of the particles for each different height, while the red continuous line to the residence time scaled by the air density. When the newly derived relationships for the variations of the winds were used (Fig. 8h), the result was similar to the one of the Hanna (1982) case.

We managed to adjust the remaining imbalance by slightly scaling the drift correction term of the Langevin equation, Eq. 5. An increase of the term by 10 $\%$ proved to be very effective. Fig. 8l shows the well-mixed test for TKEts with the addition of the constant in the drift correction term. Tuning was tested for different unstable situations and resulted in well-mixed states in the order $\pm 2\%$ throughout most of the ABL. Since the vertical slope of the $\sigma _{w}$ term is negative, the drift correction term always takes negative values. An increase of the term leads to more negative values of the term and consequently lower values of turbulent velocities. This acted to counterbalance the existing vertical gradient in the previous cases. We adopted that fix as part of the TKEts parametrization scheme.

### Simulation of Power Plant Plumes

We applied FLEXPART-COSMO in forward mode to kilometre-scale simulations of the carbon dioxide, $\mathrm {CO_2}$, plume of a large coal-fired power plant for which in situ flight observations were available for model validation (Bełchatów, Poland). Different FLEXPART runs using the three turbulence settings as discussed in the previous section were conducted. Since the power plant is located outside the COSMO-1 domain of MeteoSwiss, input fields for FLEXPART were generated by a dedicated COSMO-GHG (Jähn et al. 2020) run at a resolution of $1 \times 1 \,\hbox {km}^2$ applying similar turbulence settings as used by MeteoSwiss for operational COSMO-1 simulations. In addition to plumes simulated in FLEXPART-COSMO, COSMO-GHG was directly used to simulate the tracer concentration of the emerging $\mathrm {CO_2}$ plume, which allows us to compare Lagrangian transport in FLEXPART directly to Eulerian transport in COSMO. Both simulations considered the same vertical release profile for the emissions, which was based on initial plume rise calculations (Brunner et al. 2019). The latter assumes that plume rise happens immediately above the stack and is not spread further downwind. A detailed description of the COSMO-GHG setup, including description of the emission source and vertical release profile, is available in Brunner et al. (2021). COSMO-GHG output was stored at a temporal resolution of 15 min, which is more frequent than output in the operational MeteoSwiss chain. FLEXPART-COSMO was directly driven by these outputs releasing the same tracer mass per time unit and using the same release profile as for the COSMO-GHG tracer. A total of 2 million model particles were released over a period of 6-h.

Model simulations of $\mathrm {CO_2}$ concentrations were also compared to airborne in-situ observations carried out on-board the DLR-Cessna aircraft on 2018-06-07 during a flight consisting of an individual horizontal cross-section at a distance of 6 km from the source, and two vertical curtain flights at 12 km and 24 km from the source. Details on the observations can be found in Fiehn et al. (2020).

The meteorological conditions during the flight were highly unstable with a deep convective boundary layer reaching up to about 1700 m above ground. Winds within the boundary layer were easterly with speeds between 4 and 5 $\mathrm {m~s^{-1}}$. COSMO-GHG simulated a complex $\mathrm {CO_2}$ plume emerging from the power plant showing features of individual large turbulent eddies with diameters in the range of several kilometres (see Online Resource S20). Hence, the situation is a well suited test case for the updated turbulence description of FLEXPART.

**Fig. 9:**
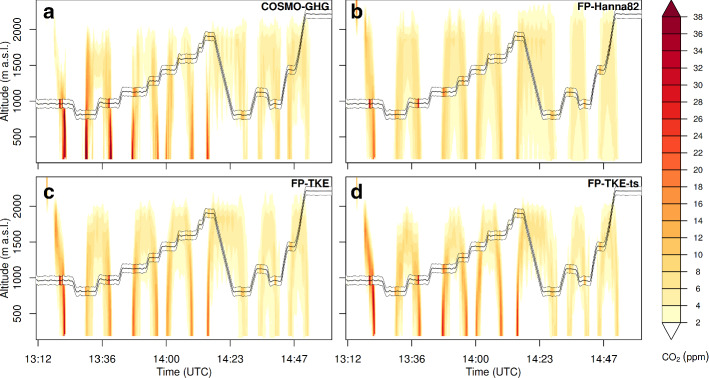
Time-height sections of simulated $\mathrm {CO_2}$ emission plume of the Bełchatów power station for 7/6/2018 along the flight track of the DLR Cessna for different transport models/configurations: **a** COSMO-GHG, **b** FLEXPART with Hanna (1982) turbulence, **c** FLEXPART with TKE-based wind variations, **d** FLEXPART with TKE-based wind variations and updated Lagrangian time scales. Observations of $\mathrm {CO_2}$ enhancements (subtracting average observations upstream from the power plant stack) are given as a band between the two black thin lines in the figures with the same colour code as the model simulations

Comparisons of the vertical plume structure along the flight path of the DLR Cessna for the two models and different FLEXPART configurations are shown in Fig. 9. It is evident that the FLEXPART simulation using the Hanna (1982) turbulence scheme largely overestimates dispersion, both in the vertical and horizontal (also compare Online Resource S20). Peaks in the FLEXPART simulated $\mathrm {CO_2}$ concentrations were generally lower than those in COSMO-GHG and those observed. Plumes were much wider and reached even beyond the top of the ABL at 1700 m a.s.l.. A direct comparison of the simulations, interpolated to the locations of the observations, reveals a regression slope of 0.6 between simulations and observations in contrast to 0.8 for COSMO-GHG (Online Resource S21). Applying the TKE-based turbulence scheme (Fig. 9c) to a certain degree improved the comparison: plume width and vertical extent were reduced, but there remained a considerable underestimation of observed concentrations at the plume centres (regression slope of 0.7). Finally, the FLEXPART simulations with TKEts (Fig. 9d) further reduced the plume dispersion. Simulated concentrations were much more comparable with those of COSMO-GHG and the observations (regression slope of 0.8).

Although this example may not be representative for all unstable situations, it clearly demonstrates that (i) FLEXPART-COSMO at kilometre-scale resolution and using the Hanna (1982) turbulence scheme is not able to represent plume dispersion at source distances covered in this comparison (smaller 25 km), (ii) adjusting the turbulence scheme strongly reduces dispersion and improves the plume representation. Despite these improvements, the comparison also indicates the limitations of offline-coupled LPDMs at this scale. Due to the limited representation of the temporal variability in the stored wind fields, FLEXPART-COSMO was not able to reproduce the complex plume structures as seen in COSMO-GHG (see Online Resource S20), but generally predicted smoother plumes.

### Simulation of $\mathrm {CH_4}$ Concentrations at the Beromünster Tall Tower

To assess the performance of the derived turbulence parametrized, one year of test simulations for $\mathrm {CH_4}$ concentrations at the Beromünster tall tower were performed with the different setups of the LPDM and compared to observations (see Sect. 2.3). The different setups consist of a FLEXPART COSMO-7 simulation, a FLEXPART-COSMO-1 simulation including offline nesting with FLEXPART-IFS, both using the Hanna (1982) turbulence scheme, and a FLEXPART-COSMO-1, using TKEts parametrized scheme, with FLEXPART-IFS (Hanna (1982) turbulence) offline nesting.

**Fig. 10:**
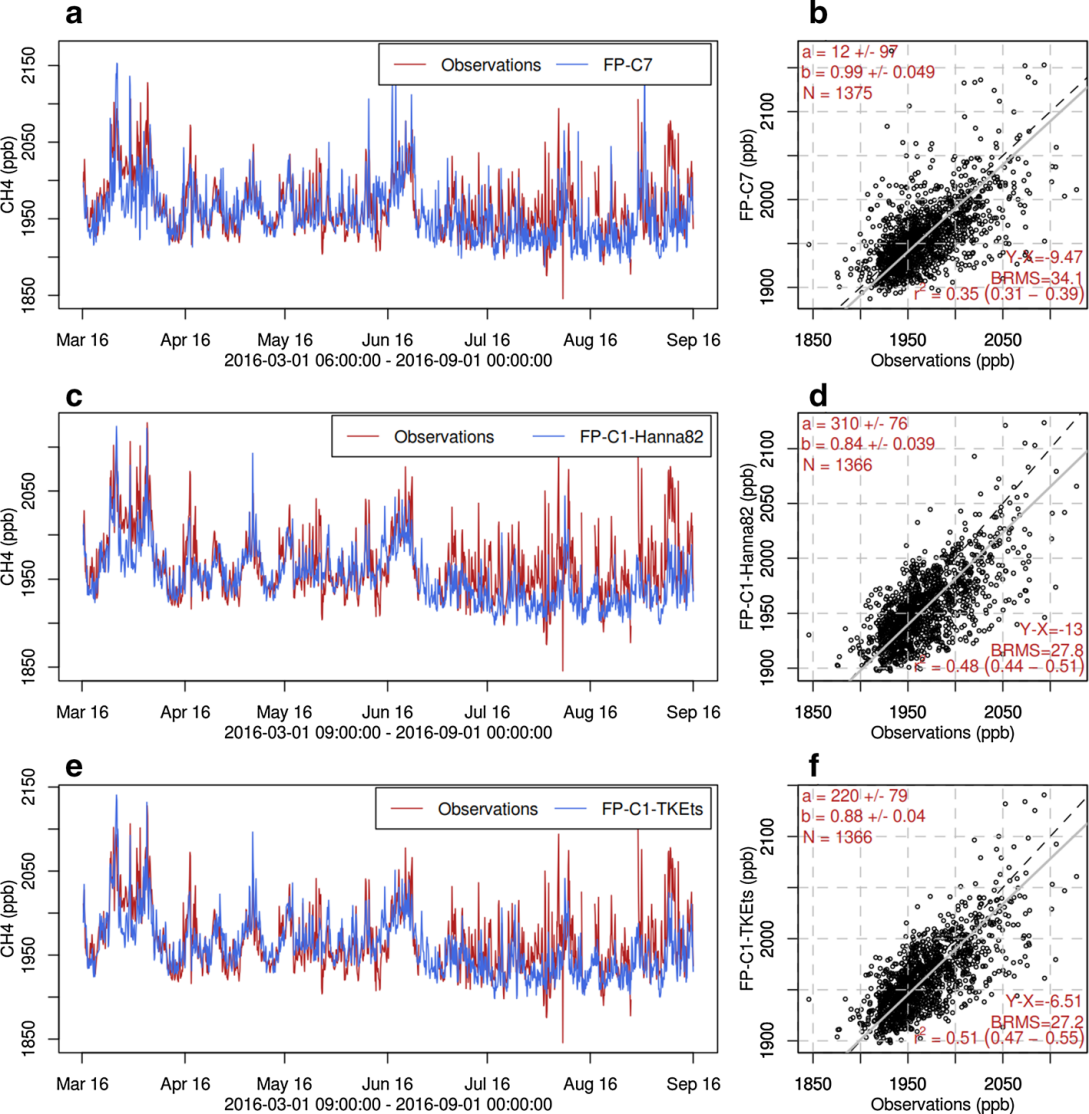
The first column shows time series of $\mathrm {CH_4}$ concentration for the period from March to September of 2016 at the receptor site in Beromünster Switzerland evaluating the **a**–**b** FLEXPART-C7, **c**–**d** FLEXPART-C1 with Hanna (1982) turbulence scheme and **e**–**f** FLEXPART-C1 with the TKEts turbulence scheme. The red lines correspond to observations, whereas blue lines represent simulations by each model configuration. The second column shows a scatter plot of observations (*x* axis) versus model (*y* axis) concentrations for all the different model configurations. The concentrations correspond to the highest inlet (212 m above ground level) of BRM tall tower. The red values in the plot correspond to: **a** regression intercept; **b** regression slope; *N*, number of data points; $Y-X$, mean bias; BRMS, bias-corrected root mean square error; $r^{2}$, coefficient of determination and its uncertainty range

The results of simulations for the period from 1 March to 17 September 2016 are summarized in Fig. 10. Note that there is a gap in the observations for the remainder of September. The focus is on the warm season because our main improvements concern unstable cases, which mainly occur during this part of the year. Time series for the whole 2016 are given in the supplement (Online Resource S22).

The high-resolution model with TKEts turbulence scheme performs significantly better than the coarse-resolution model in terms of correlation with the observations, and it is also improved when compared to its basic version (Hanna (1982) turbulence scheme). The gap between the coarse-resolution model and the high-resolution model, in terms of regression slope, was reduced considerably; FLEXPART-COSMO-1 yielded a regression slope of 0.74 before the offline coupling and the new turbulence representation. However, the low-resolution model, (a and b in Fig. 10) still outperformed the high-resolution model (e and f in Fig. 10) in terms of regression slope (0.99 vs. 0.88 for the the TKEts version of FLEXPART-C1). Similarly, normalized standard deviation (simulated divided by observed standard deviation) was closer to 1 for the low-resolution model compared to the high-resolution model, indicating that peak concentrations were still better represented. The offline coupling of FLEXPART-COSMO-1 with FLEXPART-IFS increased the concentrations by 10$\%$ when compared to the version without the offline nesting option. The introduced changes (TKEts) in the turbulence scheme and the tuning parameters further enhanced the simulated concentrations by 5$\%$ and increased the correlation of the LPDM with the observations (e–f).

**Fig. 11:**
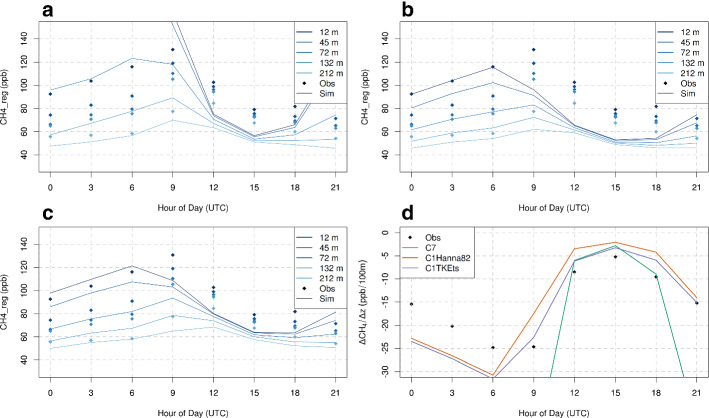
Diurnal cycle of $\mathrm {CH_{4}}$ for observations and simulated concentrations for **a** FLEXPART-COSMO-7, **b** FLEXPART-COSMO-1 Hanna (1982), and **c** FLEXPART-COSMO-1 TKEts for all the 5 inlets of Beromünster tall tower. Solid lines represent the simulated values while diamond shaped points the observations.The diurnal cycle is an average over the 6 months from March to September. Panel black values in the plot correspond shows the diurnal cycle of the vertical gradient of methane for all different model setups and observations, averaged over the same period. All values represent 3-hourly averages plotted at the end of the averaging interval (e.g., values plotted at 9 refer to the average 0600–0900 UTC)

The improvements with TKEts are also reflected in an improved representation of the diurnal cycle. This is illustrated in Fig. 11, where the low-resolution model exhibits a diurnal cycle that is shifted by 3 h when compared to observations, whereas this is not apparent in the high-resolution model with the TKEts turbulence parametrizations. It also outperforms the vertical gradient of the coarse-resolution model for most of the day. However, during daytime (0900–1500 UTC), simulated gradients were very comparable, and from 1500–1800 UTC, they were closer to the observed gradient in the coarse resolution model. For the highest inlet of the tall tower, the observations show the maximum concentration in the 3-h time window 0900–1200 UTC, whereas the low-resolution simulations produce the maximum in the time window 0600–0900 UTC. A similar pattern is discernible for the lower sampling heights for both models as well. A possible reason for this behaviour could be that the mixing increases rapidly during the early morning hours in the low-resolution model leading to a too rapid growth of the ABL. The decrease of the mixing in the high-resolution model with the new turbulence scheme seems very effective, since it leads to an improved match of the simulated and the observed diurnal cycles (Fig. 11).

This is further illustrated in Fig. 11d showing the diurnal cycle of the vertical gradient of $\mathrm {CH_4}$. The observed gradient was most negative during the early morning hours, when values of $-25\,\hbox {ppb} / 100\, \hbox {m}$ were reached. During the convective part of the day the gradient became less negative ($-5\,\mathrm{{ppb}}/100\,\mathrm{{m}}$). Due to the reduced vertical mixing in the TKEts turbulence scheme, higher absolute values of the vertical gradient result when compared to the Hanna (1982) version. In fact, the simulated vertical gradient is closer to the one seen in the observations for the whole day in the LPDM with the TKEts turbulence scheme. It also outperforms the vertical gradient of the coarse-resolution model except for the time window 0900–1200 UTC where the coarse resolution model is closer to the observations.

In previous studies where FLEXPART-COSMO-7 was used for inverse modelling and $\mathrm {CH_4}$ emissions estimation, only the top inlet of BRM was utilized and only the afternoon hours of each day were considered in the inversion. The introduction of FLEXPART-COSMO-1 for inverse modelling studies could potentially use observations from all five sampling heights at BRM, since the model performs well at all heights. Observations from the whole day, and not only afternoon values, could also be considered, since the diurnal cycle of the vertical gradient of the tracer does not differ significantly when compared to the observations, in contrast to the coarse resolution model.

We present additional details on the performance differences between the high- and low-resolution model in the form of Taylor plots (Online Resource 23). The statistical measures used in the Taylor plot are the correlation, the normalized standard deviation–which is the standard deviation of the simulation divided by the standard deviation of the observations–and the model skill score as discussed in Taylor (2001). The high-resolution model with TKEts outperforms the low-resolution model in almost all the different measures utilized and for all different sampling heights. For the inlets at 132 m and 212 m of the tall tower the two models present similar variabilities, but the high-resolution model is significantly better in terms of correlation and model skill, something which was already demonstrated in Figs. 10 and 11 . For simulations for the lower three sampling heights ($\le$72 m) the low-resolution model largely overestimates observed variability and/or shows poor correlations. In contrast, the high-resolution model performs almost equally well at all different vertical levels. Part of the large difference in the statistics of the coarse-resolution model with height may arise from the less detailed representation of the topography in COSMO-7, which results in an elevation deficit of approximately 190 m at BRM compared to 80 m in COSMO-1. Online resource (23b) shows the same measures as before for the high- and the coarse-resolution model, for the highest sampling height (212 m) separately for each month of 2016. Finally, when focusing on individual months, the high resolution model at 212 m again showed improved performance compared to the coarse resolution model for all months, except for October and November (Online Resource 23b). The latter could be attributed to the difficulty of COSMO-1 to simulate long lasting low stratus clouds (high fog) events (Westerhuis et al. 2020), which are typical for the Swiss Plateau during the fall and winter months.

## Conclusions

This study highlights the importance of a resolution-dependent turbulence representation in high-resolution LPDMs and introduces a practical way to adjust the turbulence scheme of any LPDM to follow the parameterized turbulence produced by the driving NWP. Although, the proposed turbulence scheme was derived specifically for the used COSMO-1 configuration, it should still be applicable by any LPDM driven with NWP data at similar resolution.

We first analyzed the influence of model resolution on greenhouse gas concentrations at a tall tower site in Switzerland for the FLEXPART-COSMO LPDM. When moving from a $7\times 7\,\hbox {km}^2$ to a $1 \times 1 \,\hbox {km}^2$ horizontal resolution in COSMO, we observed a distinct underestimation of the measured concentration enhancements above background.

Since the domain size of COSMO-1 is much smaller in comparison to COSMO-7, we coupled FLEXPART-COSMO-1 to FLEXPART-IFS to allow for a similar integration domain. This in itself increased the simulated concentrations in FLEXPART-COSMO-1 at the receptor site by approximately 10$\%$. This clearly indicates that for similar simulations of LPDMs in the European domain and for simulation time windows of 5–10 days, the inclusion of a larger continental domain is beneficial, since non-local (continental scale) emission sources significantly contribute to regional concentrations at the receptor location, at least when the sources are widespread as in the case of $\mathrm {CH_4}$.

Comparisons of wind variability (turbulence) resolved on the grid-scale and subgrid turbulence as predicted and diagnosed by the 1.5-order turbulence schemes of COSMO and FLEXPART, respectively, suggest that additional dispersion differences originate from a duplication of turbulent transport at high model resolution in FLEXPART: on the one hand, covered by the high resolution grid of the Eulerian model and, on the other hand, diagnosed by FLEXPART’s turbulence scheme.

A possible solution to the duplication of turbulence was found by introducing of new equations for the variations of the winds in FLEXPART mimicking the TKE predicted by the turbulence scheme of COSMO and, hence, only treat the unresolved part of turbulence in the LPDM. Additional considerations were needed regarding the Lagrangian time scales, since they are formulated as functions of the variations of the winds in FLEXPART.

We showed that the new equations for the variations of the winds together with scaled Lagrangian time scales have a profound impact on the mixing of a tracer in the ABL. Simulations with the newly derived turbulence scheme showed significant improvements in the high-resolution model’s ability to predict the vertical profiles of the observed $\mathrm {CH_4}$ variability and concentrations at the Beromünster tall tower. The diurnal cycle of the tracer for the period of reference was also significantly improved and mimicked the observed diurnal cycle at the receptor. In addition, power plant plume simulations employing the high resolution model with the new turbulence scheme showed remarkable improvements in the plume representation as compared to employing the old Hanna (1982) scheme. The latter simulations also show that FLEXPART-COSMO compared to COSMO itself was clearly more dispersive with the default Hanna (1982) scheme, whereas the agreement was improved with the new scheme. Thus, the increased dispersion cannot be attributed solely to COSMO wind fields.

Turbulence schemes of LPDMs were built to operate with coarse-resolution models in which turbulence is entirely a subgrid process. With increasing resolution of the weather prediction model driving the LPDM, part of the turbulence spectrum becomes grid-resolved. One possibility is the direct use of TKE calculated by the Eulerian model (Verreyken et al. 2019). Here, such a link was established indirectly, and has the advantage of building on a well-established framework for the integration of the Langevin equation. In addition to the Hanna (1982) scheme, the latest main version of FLEXPART includes an alternative Langevin equation for representing skewed turbulence distributions in convective boundary layers (Cassiani et al. 2015; Pisso et al. 2019). However, the computational costs for this scheme are at least 2.5 times larger than for the Hanna (1982) scheme (Pisso et al. 2019), which we deemed excessive for simulations covering long periods. In any case, the derived equations for the variations of the winds depend on the NWP model utilized to drive the LPDM. If the turbulence scheme of the NWP model either underestimates or overestimates TKE, this would have a direct impact onto the dispersion in the LPDM as well. Furthermore, these equations are derived for a region around Beromünster, Switzerland, and even if they proved to be valid at other locations in our model domain, it would be a generalisation to say that they have universal validity if the models would be applied in a different domain or with different settings. Moreover, we chose a simple scaling approach for the Lagrangian time scales as we did not establish any other robust way to calculate them directly. Furthermore, we focused on the representation of parameterized TKE in FLEXPART-COSMO as the main source for increased dispersion when operated at high resolution, while there may be additional effects that are related to topography. On the one hand, increased grid scale turbulence is partly related to an improved representation of topography, but on the other hand, horizontal turbulence may be misattributed to vertical motion due to steeper model slopes at finer resolution.

In our current study we did not explicitly treat mesoscale turbulence as an additional part of the wind vector, since its contribution to dispersion at the resolved scales (kilometres and hourly) and using FLEXPART’s default parametrization for mesoscale turbulence was small. Other authors have suggested that even at the model scales addressed here mesoscale turbulence is still considerable and should not be neglected (Webster et al. 2018). Future research and validation studies are needed to clarify the interaction of different types of motions at scales where stochastic turbulence and mesoscale features overlap.

Future work should focus on applying the proposed method using another NWP model as a driver in order to test the method’s validity. This is especially true for NWP models in the grey zone of turbulence, and for different turbulence schemes (e.g., outputs of LES) (Cornwell et al. 2021). In this context,validation of TKE as predicted in NWP against LES would be beneficial to assure that turbulence is represented adequately in the driving models. Equations for the variations of the winds with model resolution dependency and universal validity could be another focus of a future study together with a robust way of calculating the Lagrangian time scales for the part of the turbulence spectrum that needs to be resolved.

## Supplementary Information

Below is the link to the electronic supplementary material.

Supplementary file 1 (pdf 5707 KB)

## Data Availability

The data that support the findings of this study are available on request from the corresponding author. The numerical model simulations upon which this study is based are too large to be publicly archived.
