# Supplementary material for: Lagrangian Particle Dispersion Models in the Grey Zone of Turbulence: Adaptations to FLEXPART-COSMO for Simulations at 1 km Grid Resolution
Source: Boundary Layer Meteorol. 2022 Aug 5;185(1):129–60. doi: 10.1007/s10546-022-00728-3 (PMC9463295; doi:10.1007/s10546-022-00728-3)
Supplement: Supplementary file 1 — (pdf 5707 KB) [file 10546_2022_728_MOESM1_ESM.pdf]

Supplement

Ioannis Katharopoulos

June 29, 2022

S-1 Figures

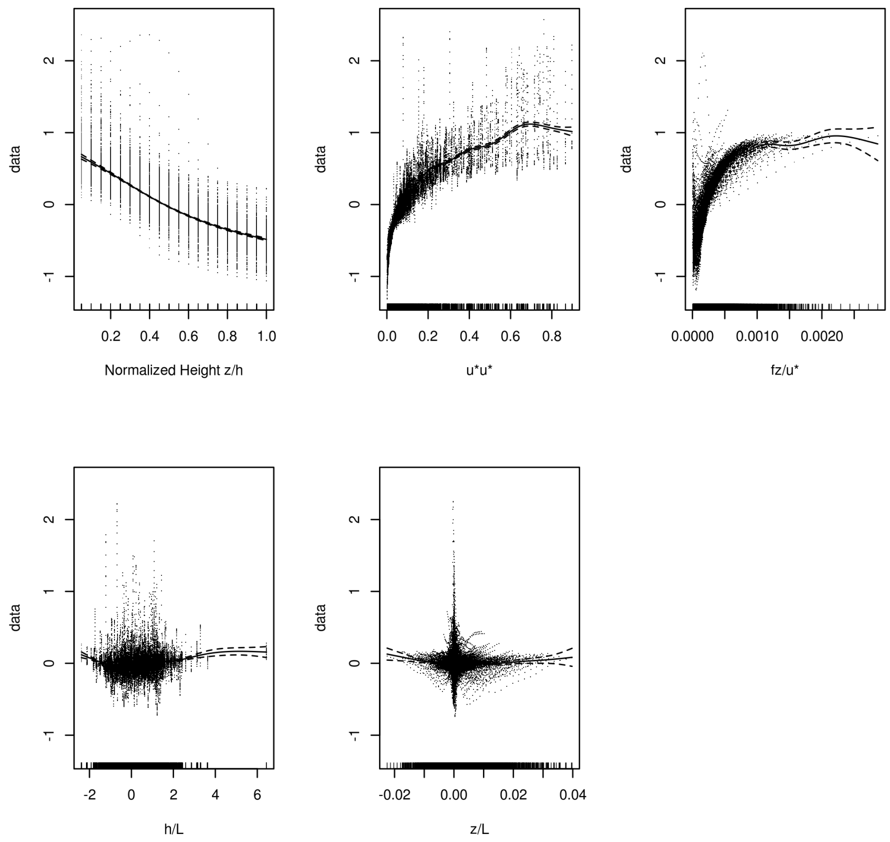

Figure S1: Plot of the response against the predictor variables $\frac{z}{h}$, u_*^2, $\frac{fz}{u_*}$, $\frac{h}{L}$, $\frac{z}{L}$ from GAM model for neutral stability.

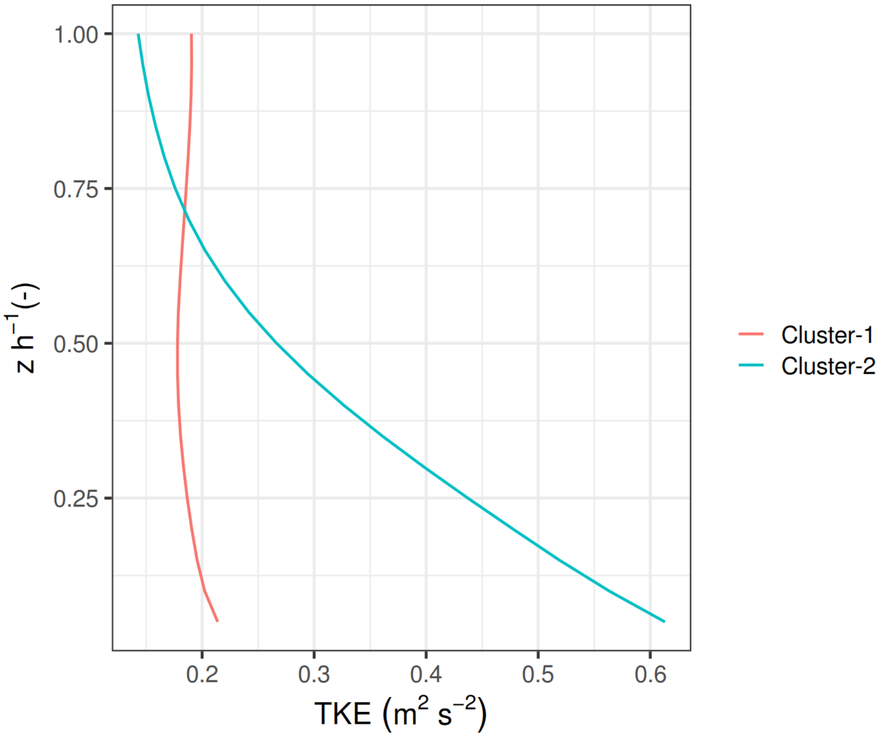

Figure S2: The two dominant vertical TKE profiles during unstable conditions.

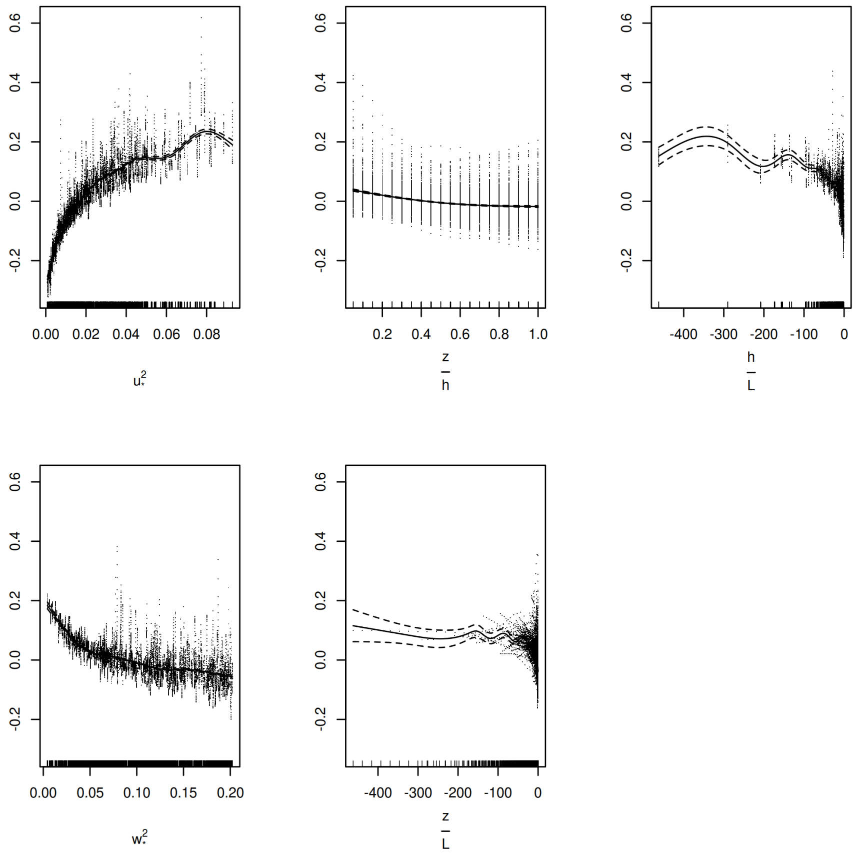

Figure S3: Plot of the response against the predictor variables (u_*^2 , $\frac{z}{h}$, $\frac{h}{L}$, w_*^2, $\frac{z}{L}$) from GAM model for cluster-1 unstable conditions.

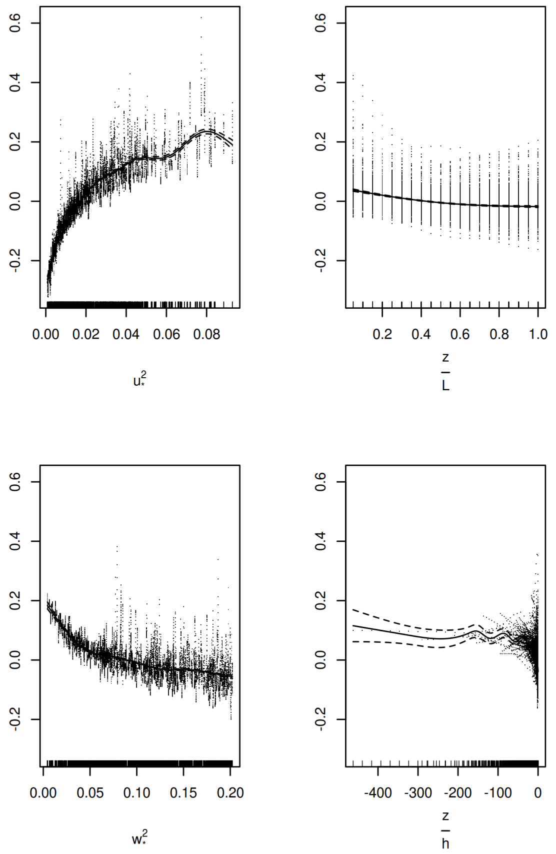

Figure S4: Plot of the response against the predictor variables (u_*^2 , $\frac{z}{h}$, $\frac{h}{L}$, w_*^2, $\frac{z}{L}$) from GAM model for cluster-2 unstable conditions.

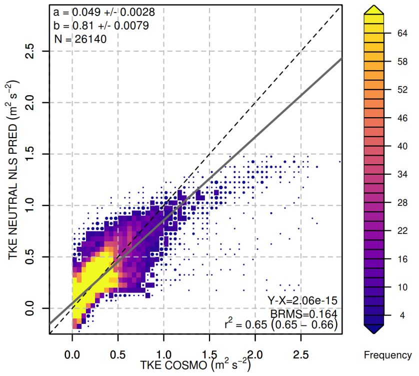

Figure S5: Scatter plot of the TKE values between the GAM model estimates (y axis) and the COSMO values (x axis) for neutral cases

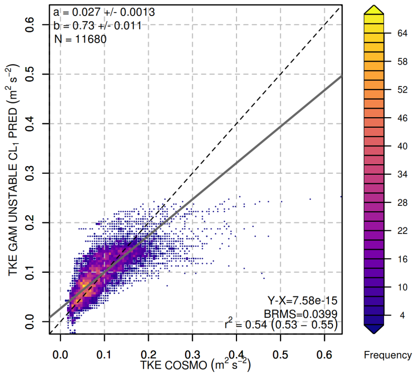

Figure S6: Scatter plot of the TKE values between the GAM model estimates (y axis) and the COSMO values (x axis) for cluster 1 unstable cases

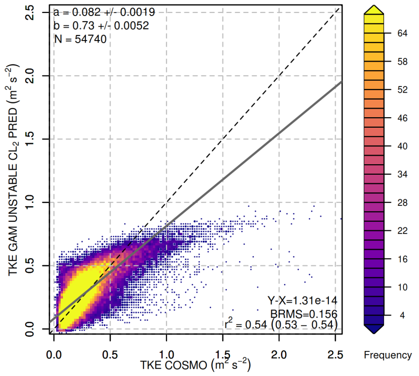

Figure S7: Scatter plot of the TKE values between the GAM model estimates (y axis) and the COSMO values (x axis) for cluster 2 unstable cases

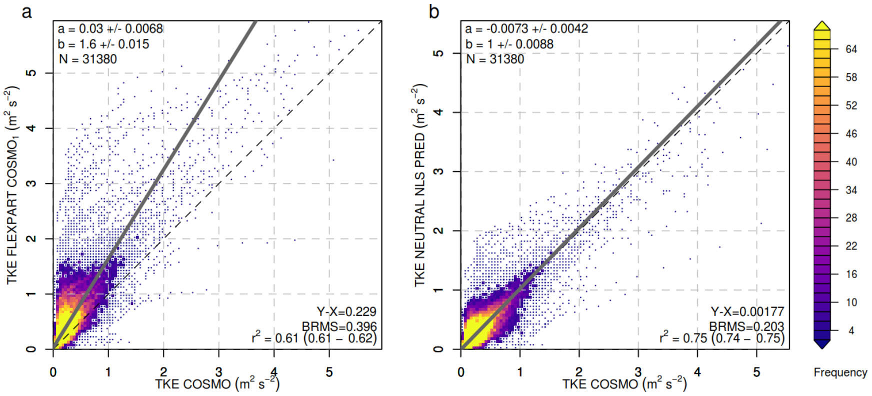

Figure S8: Density scatter plots of the TKE values between the COSMO predictions (x axis) and FLEXPART diagnosed TKE (y-axis) for (a) the Hanna82 scheme and (b) the new scheme proposed in this study, both during neutral stability for Munich region. Colours represent the number of data points (frequency) in a given grid area. Regression slopes were calculated through weighted least-square regression.

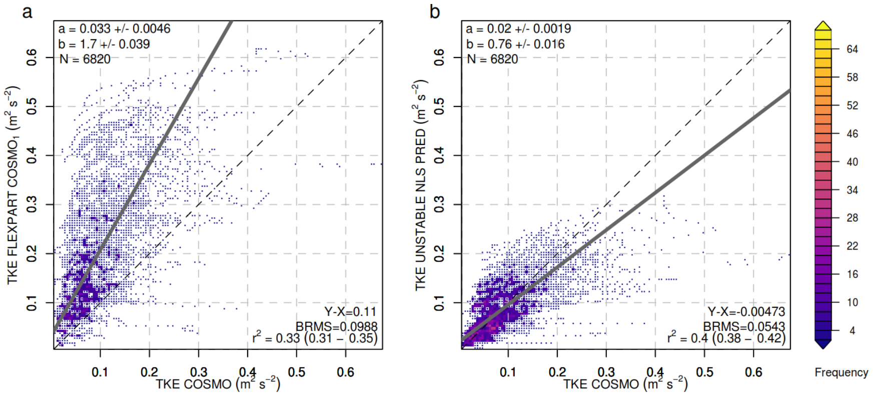

Figure S9: Density scatter plots of the TKE values between the COSMO predictions (x axis) and FLEXPART diagnosed TKE (y-axis) for (a) the Hanna82 scheme and (b) the new scheme proposed in this study, both during cluster-1 unstable cases for Munich region. Colours represent the number of data points (frequency) in a given grid area. Regression slopes were calculated through weighted least-square regression.

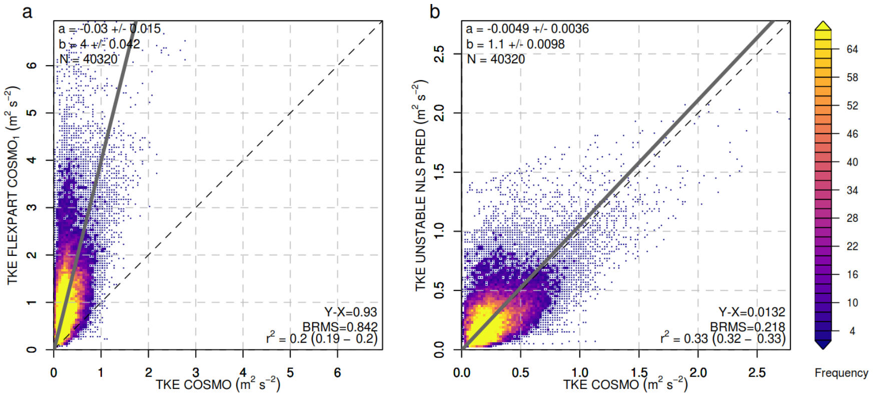

Figure S10: Density scatter plots of the TKE values between the COSMO predictions (x axis) and FLEXPART diagnosed TKE (y-axis) for (a) the Hanna82 scheme and (b) the new scheme proposed in this study, both during cluster-2 unstable cases for Munich region. Colours represent the number of data points (frequency) in a given grid area. Regression slopes were calculated through weighted least-square regression.

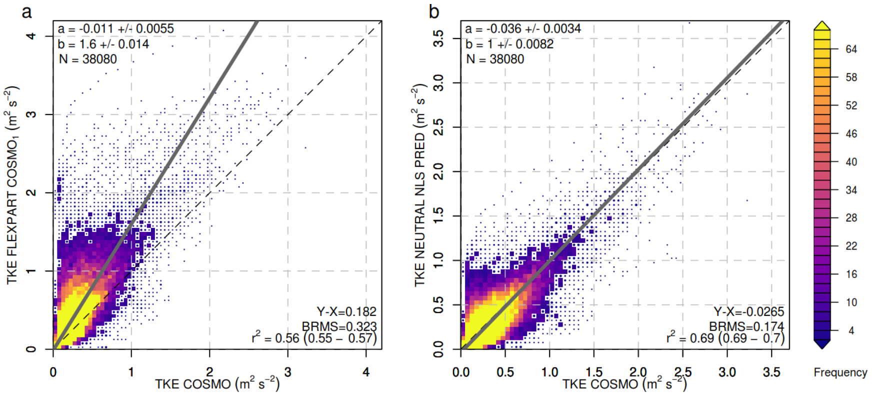

Figure S11: Density scatter plots of the TKE values between the COSMO predictions (x axis) and FLEXPART diagnosed TKE (y-axis) for (a) the Hanna82 scheme and (b) the new scheme proposed in this study, both during neutral stability for a flat area in Eastern France. Colours represent the number of data points (frequency) in a given grid area. Regression slopes were calculated through weighted least-square regression.

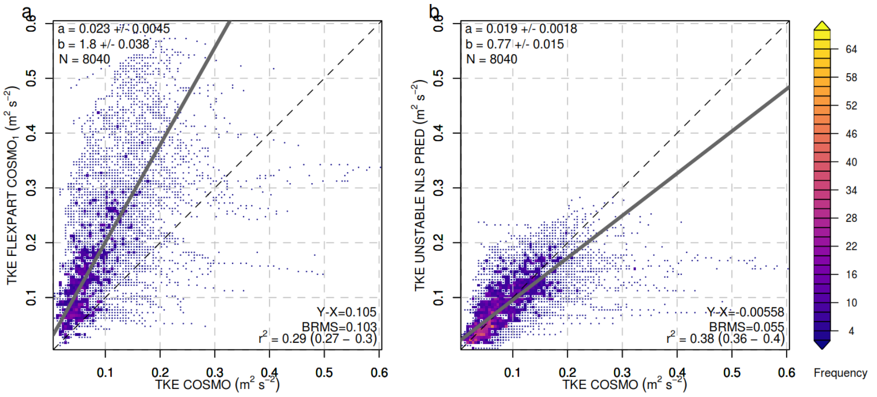

Figure S12: Density scatter plots of the TKE values between the COSMO predictions (x axis) and FLEXPART diagnosed TKE (y-axis) for (a) the Hanna82 scheme and (b) the new scheme proposed in this study, both during cluster-1 unstable cases for a flat area in Eastern France. Colours represent the number of data points (frequency) in a given grid area. Regression slopes were calculated through weighted least-square regression.

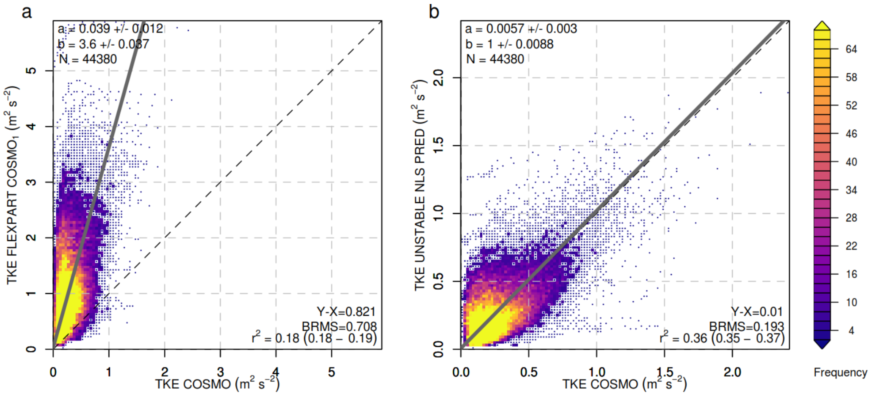

Figure S13: Density scatter plots of the TKE values between the COSMO predictions (x axis) and FLEXPART diagnosed TKE (y-axis) for (a) the Hanna82 scheme and (b) the new scheme proposed in this study, both during cluster-2 unstable cases for a flat area in Eastern France. Colours represent the number of data points (frequency) in a given grid area. Regression slopes were calculated through weighted least-square regression.

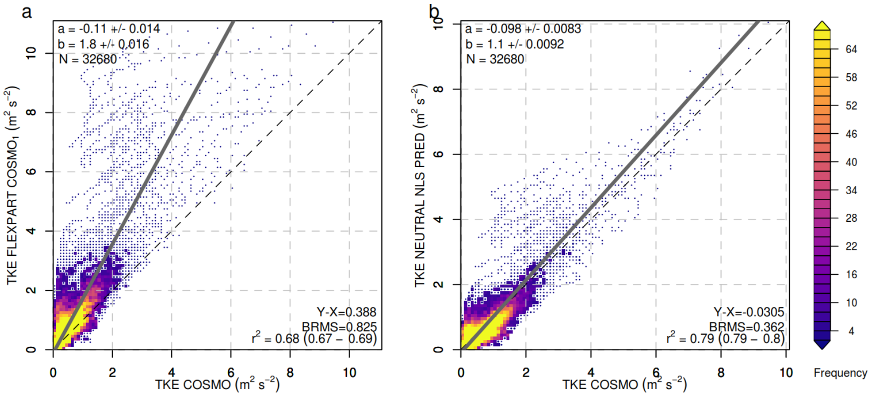

Figure S14: Density scatter plots of the TKE values between the COSMO predictions (x axis) and FLEXPART diagnosed TKE (y-axis) for (a) the Hanna82 scheme and (b) the new scheme proposed in this study, both during neutral stability in St. Gallen. Colours represent the number of data points (frequency) in a given grid area. Regression slopes were calculated through weighted least-square regression.

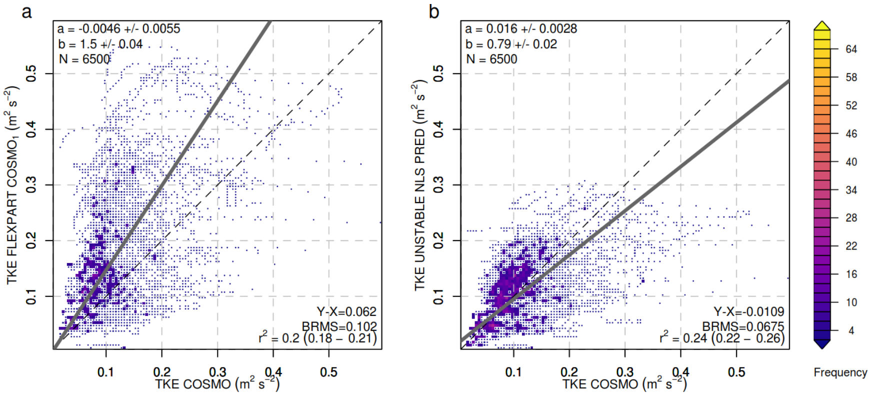

Figure S15: Density scatter plots of the TKE values between the COSMO predictions (x axis) and FLEXPART diagnosed TKE (y-axis) for (a) the Hanna82 scheme and (b) the new scheme proposed in this study, both during cluster-1 unstable cases. Colours represent the number of data points (frequency) in a given grid area. Regression slopes were calculated through weighted least-square regression.

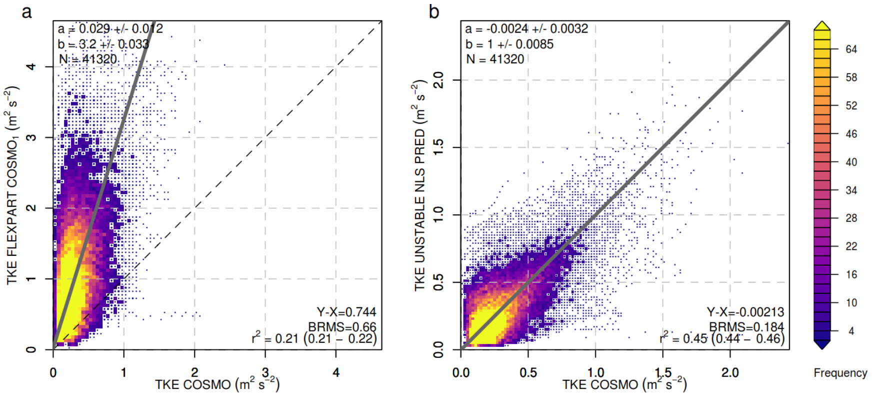

Figure S16: Density scatter plots of the TKE values between the COSMO predictions (x axis) and FLEXPART diagnosed TKE (y-axis) for (a) the Hanna82 scheme and (b) the new scheme proposed in this study, both during cluster-2 unstable cases in St. Gallen. Colours represent the number of data points (frequency) in a given grid area. Regression slopes were calculated through weighted least-square regression.

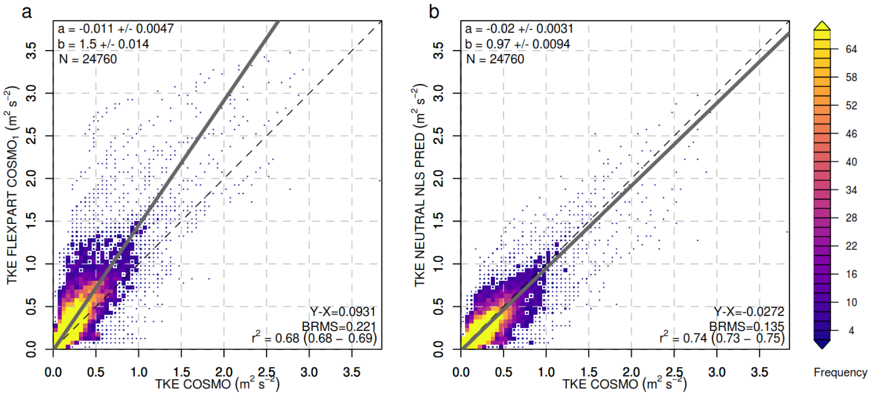

Figure S17: Density scatter plots of the TKE values between the COSMO predictions (x axis) and FLEXPART diagnosed TKE (y-axis) for (a) the Hanna82 scheme and (b) the new scheme proposed in this study, both during neutral stability in Po Valley in North Italy. Colours represent the number of data points (frequency) in a given grid area. Regression slopes were calculated through weighted least-square regression.

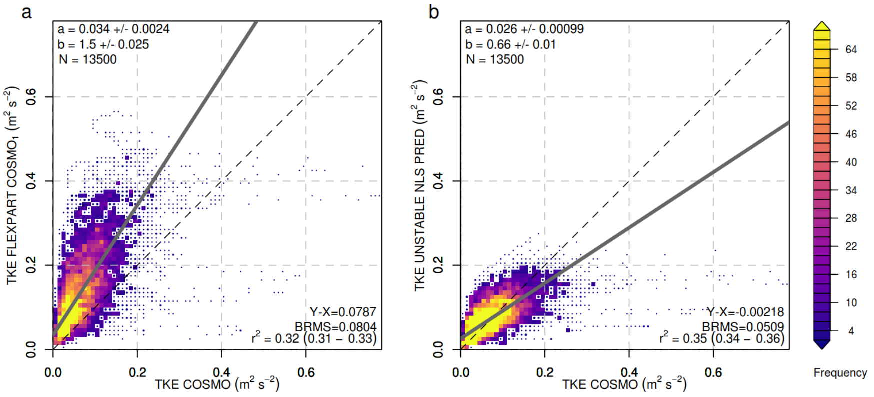

Figure S18: Density scatter plots of the TKE values between the COSMO predictions (x axis) and FLEXPART diagnosed TKE (y-axis) for (a) the Hanna82 scheme and (b) the new scheme proposed in this study, both during cluster-1 unstable cases in Po Valley in North Italy. Colours represent the number of data points (frequency) in a given grid area. Regression slopes were calculated through weighted least-square regression.

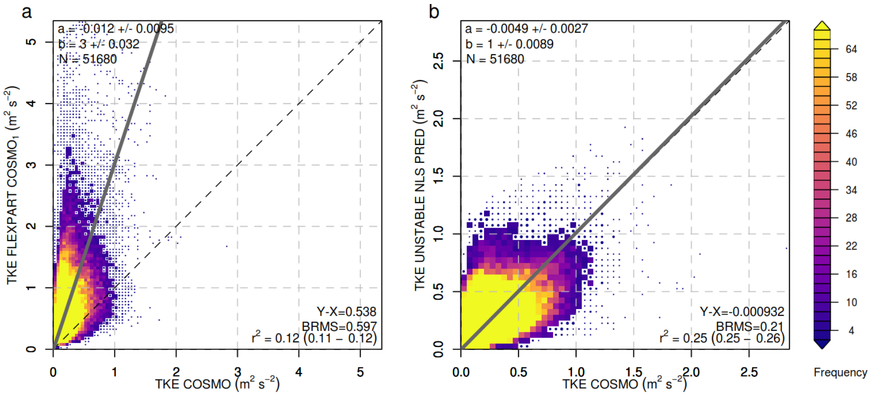

Figure S19: Density scatter plots of the TKE values between the COSMO predictions (x axis) and FLEXPART diagnosed TKE (y-axis) for (a) the Hanna82 scheme and (b) the new scheme proposed in this study, both during cluster-2 unstable cases in Po Valley in North Italy. Colours represent the number of data points (frequency) in a given grid area. Regression slopes were calculated through weighted least-square regression.

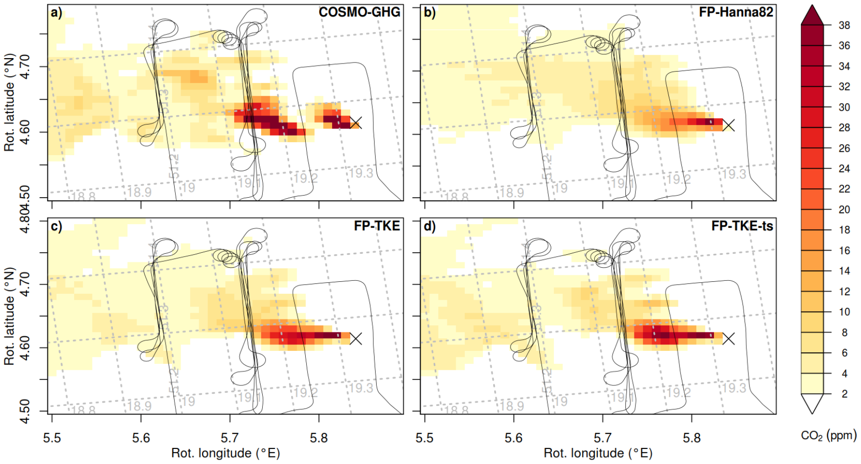

Figure S20: Horizontal distribution of simulated CO_2 power plant plume of the Bełchatów power station (cross) for 2018-06-07 13:30 UTC and different transport models/configurations: a) COSMO-GHG, b) FLEXPART with Hanna82 turbulence, c) FLEXPART with TKE-based wind variations, d) FLEXPART with TKE-based wind variations and updated Lagrangian timescales. Shown are mole fractions at model level 8 at ∼600 m a.s.l. The flight track of the DLR Cessna is shown as a thin black line. Flight direction was from east to west.

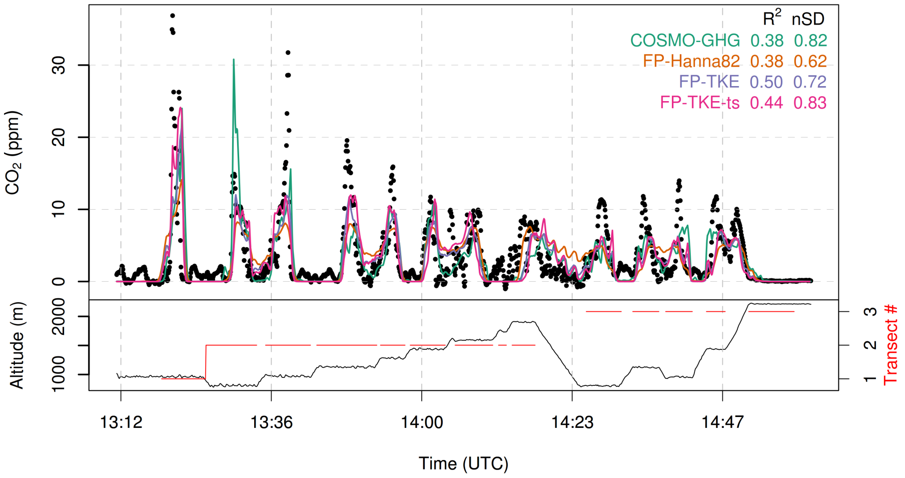

Figure S21: Time series comparison of CO_2 concentration for all the different model representations (continuous lines) and the observations (points) during the flight at the Belchatow power station for 2018-06-07. In the topright part of the figure the correlation of each model representation with the observations and the normalised standard deviation between the model and the observations are summarised. The lower panel shows the altitude of the airplane during the flight.

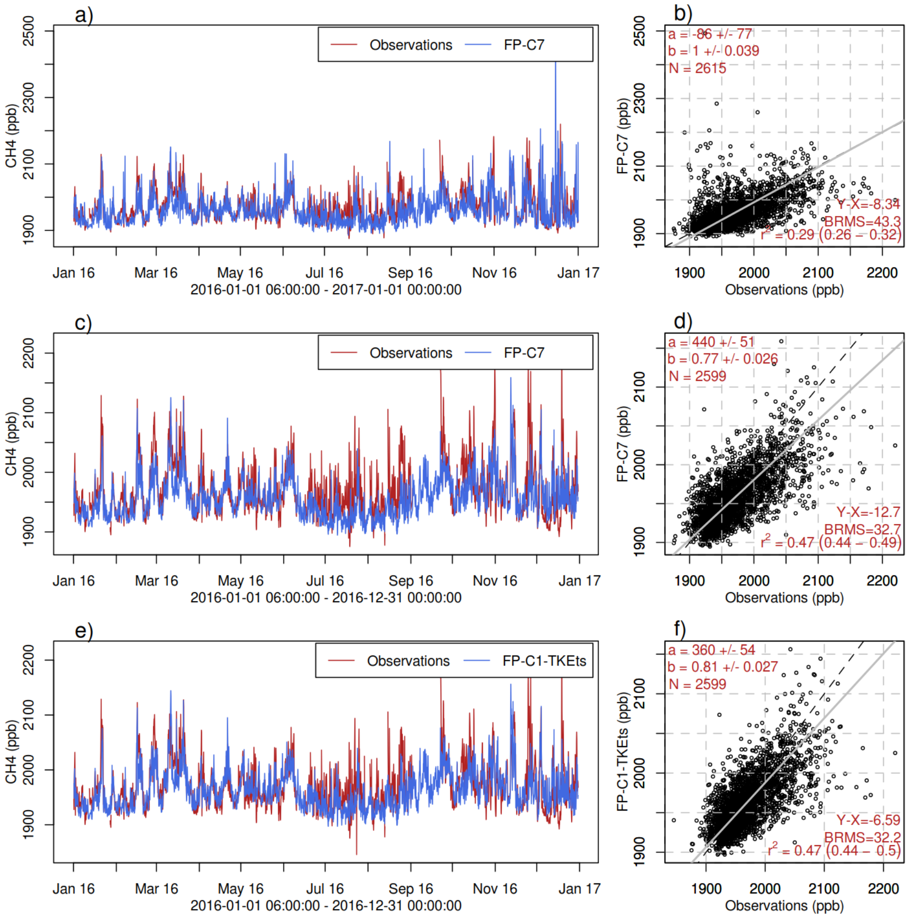

Figure S22: The first column shows time series of CH_4 concentration for the period from March to September of 2016 at the receptor site in Beromünster Switzerland evaluating the (a-b) FLEXPART-C7, (c-d) FLEXPART-C1 with Hanna82 turbulence scheme and (e-f) FLEXPART-C1 with the newly derived parameterizations turbulence scheme. Red line corresponds to observations, whereas blue lines represent simulations by each model configuration. The second column shows a scatter plot of observations (x axis) vs model (y axis) concentrations for all the different model configurations. The concentrations correspond to the highest inlet (212 m above ground level) of BRM tall tower and cover the year 2016. .

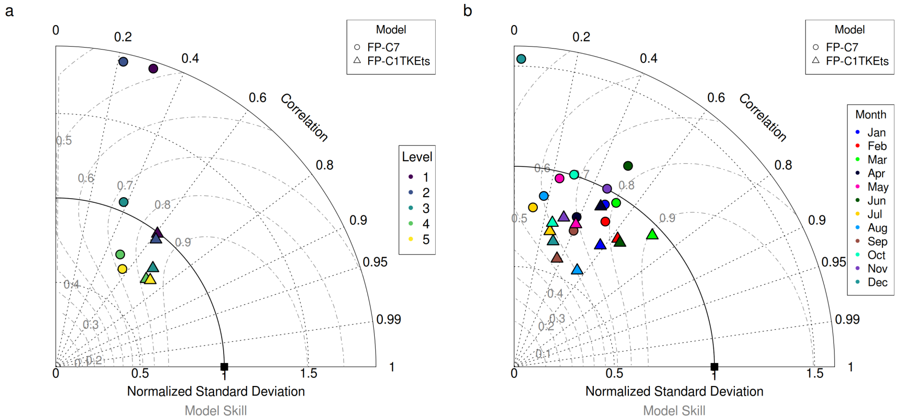

Figure S23: a) Taylor plot showing correlation, normalised standard deviation and model skill between simulation and observations for the high resolution model with the new parameterisation scheme and the coarse resolution model for the 6 months from March to September of 2016 and for all different sampling heights at BRM and b) for individual months for the highest sampling height (212 m) at BRM. Each sampling height in a) and month in b) is depicted by a different colour. The circles correspond to the coarse resolution model while the triangles to the high resolution model.

S-2 Equations

The following equations are the derivatives of the vertical component of the variations of the wind for neutral and unstable conditions. They correspond to the equations introduced in Section 3.1 of the main manuscript and are required for the drift correction term of the Langevin equation.

- **Neutral Conditions**

$$\frac{\partial \sigma_w}{\partial z} = \frac{\left(-4\beta f u_* \exp\left(\frac{-4fz}{u_*}\right) + \frac{\gamma \delta u_*^2 \exp\left(\frac{\delta z}{h}\right)}{h}\right)}{2\sigma_w} \tag{1}$$

- **Unstable conditions, cluster-1** ($w_* < 0.45$)

$$\frac{\partial \sigma_w}{\partial z} = \frac{\beta}{\sigma_w}\left(\frac{1.2\gamma w_*^2}{h}\left(1 + \frac{\gamma z}{h}\right) - \delta\lambda \frac{u_*^2}{h}\left(1.81 - \lambda \frac{z}{h}\right)\right) \tag{2}$$

- **Unstable conditions, cluster 2** ($w_* \geq 0.45$)

$$\frac{\partial \sigma_w}{\partial z} = \frac{\beta \gamma w_*^2 \exp\left(\frac{\gamma z}{h}\right) + \delta \lambda u_*^2 \exp\left(\frac{\lambda z}{h}\right)}{2h\sigma_w} \tag{3}$$
